# Enhanced fatty acid availability rewires fibroblasts metabolism and decreases collagen deposition in lung fibrosis

**DOI:** 10.1007/s00018-026-06256-4

**Published:** 2026-06-01

**Authors:** Gabriela Wojcik, Anita Sahu-Osen, Pamela Waked, Juergen Gindlhuber, Ayu Hutami Syarif, Andelko Hrzenjak, Chandran Nagaraj, Anna Birnhuber, Julia Hoffmann, Lena Mischkulnig, Ehsan Bonyadirad, Malgorzata Wygrecka, Clemens Aigner, Alberto Benazzo, Andrea Olschewski, Sander Kersten, Saverio Bellusci, Leigh M. Marsh, Elie El Agha, Paul Vesely, Grazyna Kwapiszewska, Valentina Biasin

**Affiliations:** 1 Otto Loewi Research Centre, Lung Research Group, Medical University of Graz, Graz, Austria; 2 Ludwig Boltzmann Institute for Lung Vascular Research, Graz, Austria; 3 Otto Loewi Research Centre, Division of Physiology and Pathophysiology, Medical University of Graz, Neue Stiftingtalstrasse 6/V, Graz, 8010 , Austria; 4 Division of Cardiology, University Heart Centre Graz, Medical University of Graz, Graz, Austria; 5 Department of Internal Medicine, Division of Respiratory Medicine, Medical University of Graz, Graz, Austria; 6 Lung Research Cluster, Medical University of Graz, Graz, Austria; 7 Michael Popp Institute, Centre for Molecular Biosciences Innsbruck (CMBI), University of Innsbruck, Innsbruck, Tirol Austria; 8 Institute for Lung Health (ILH), Giessen, Germany; 9 Department of Medicine II, Internal Medicine, Pulmonary and Critical Care, Universities of Giessen and Marburg Lung Center (UGMLC), German Center for Lung Research (DZL), Justus-Liebig-University Giessen, Giessen, Germany; 10 Department of Thoracic Surgery, Comprehensive Centre for Chest Diseases, Medical University of Vienna, Vienna, Austria; 11 Otto Loewi Research Center, Medical University of Graz, Graz, Austria; 12 Nutrition, Metabolism and Genomics Group, Division of Human Nutrition and Health, Wageningen University, Wageningen, The Netherlands; 13 Cardio-Pulmonary Institute (CPI), Giessen, Germany; 14 Department of Medicine V, Internal Medicine, Infectious Diseases and Infection Control, Universities of Giessen and Marburg Lung Centre (UGMLC), German Centre for Lung Research (DZL), Justus-Liebig University Giessen (JLU), Giessen, Germany; 15 Diagnostic and Research Institute of Pathology, Medical University of Graz, Graz, Austria; 16 BioTechMed-Graz, Graz, Austria

**Keywords:** Fibroblasts phenotype, Lung fibrosis, Fatty acids, Collagen

## Abstract

Pulmonary fibrosis is characterized by irreversible remodeling of the lung parenchyma, which leads to a progressive deterioration in lung function. Pro-fibrotic fibroblasts contribute to uncontrolled extracellular matrix deposition and progression of fibrosis. Emerging evidence implicates metabolic dysregulation - particularly enhanced glycolysis and impaired fatty acid metabolism - in the pathogenesis of pulmonary fibrosis. Here, we investigated whether modulating fatty acid metabolism could counteract fibroblast-driven fibrosis in experimental models. Treatment with pharmacological agents (metformin, rosiglitazone), or genetic ablation of the LPL inhibitory protein, angiopoietin like 4, markedly reduced lung fibrosis and ECM production in bleomycin-induced lung fibrosis mouse model. Similarly, in vitro, provision of albumin-bound fatty acids (FA) in the growth medium reduced TGFβ1-induced collagen production in lung fibroblasts. Mechanistically, excess fatty acid reduced glycolysis, while enhancing mitochondrial function and overall metabolic activity in TGFβ1-treated fibroblasts. This metabolic shift, but not the collagen reduction, was driven by increased fatty acid oxidation via CPT1, as confirmed by the use of the CPT1 inhibitor etomoxir. Importantly, in vivo metabolic interventions downregulated preferentially PDGFRα protein level, while in vitro provision of albumin-bound FA reduced both the amount of collagen produced by αSMA-positive cells and the amount of PDGFRα-positive cells, suggesting a dual role of FA on both pro-fibrotic fibroblasts. Collectively, our findings identify fatty acid oxidation as a potent metabolic checkpoint in fibrotic fibroblasts and support FA availability as a promising strategy to limit lung fibrosis progression.

**Supplementary Information:**

The online version contains supplementary material available at 10.1007/s00018-026-06256-4.

## Introduction

Idiopathic pulmonary fibrosis (IPF) is a fatal disease characterized by massive scarring of the lung tissue eventually leadings to respiratory failure [1, 2]. Lung injury, along with genetic predisposition and environmental factors are thought to be the main causes of IPF [3–7]. The only drugs approved for treatment, pirfenidone and nintedanib, slow the progression of the disease, but cannot prevent extensive tissue remodeling and halt further progression [8–10]. The molecular hallmarks of IPF are inflammation, fibroblast proliferation and massive deposition of interstitial extracellular matrix (ECM) [11–13]. Different types of fibroblasts have been shown to take part in ECM production such as platelet-derived growth factor receptor alpha-positive (PDGFRα+) fibroblasts and alpha-smooth muscle actin-positive fibroblasts (ACTA2+; myofibroblasts) [14].

The lung is metabolically a highly-active tissue [15] in which the balance between glycolysis and β-oxidation contributes to the maintenance of essential biological processes and tissue integrity [15]. In lung fibrosis, fibroblasts maintain an elevated glycolytic activity [16] with elevated lactate production [17, 18]. This metabolic rewiring sets up a vicious cycle, as it is not only responsible for the trans-differentiation of fibroblasts into pro-fibrotic myofibroblasts [13, 19], but it also directly affects ECM by enhancing latent TGFβ1 activation [20] and collagen production [21].

Fatty acids (FA) are the essential building blocks of lung surfactant phospholipids [22, 23] and fuel mitochondrial β-oxidation mediated ATP production [23]. Several studies have reported differences in circulating and lung tissue FA content between IPF patients and healthy donors [24, 25]. Palmitic acid, oleic acid and stearic acid, most common FA in adults serum [26], are altered in lung fibrosis [25, 27]. Indeed, decreased levels of palmitic and oleic acids have been associated with impaired surfactant function in IPF [25], while stearic acid has been shown to reduce TGFβ1-induced collagen production [27]. Interestingly, rosiglitazone and metformin, two drugs used to target insulin resistance and lipid metabolism [28, 29] have been shown to reduce pulmonary fibrosis and collagen production in preclinical models [30–32]. Similarly, the absence of angiopoietin like 4 (ANGPTL4), an endogenous inhibitor of lipoprotein lipase (LPL), has been reported to suppress bleomycin induced lung fibrosis in mice [33]. Although these three metabolic interventions target different pathways, they all reduce lung fibrosis [34–36].

In this study, we hypothesized that enhancing FA availability in lung fibroblasts induces fatty acid oxidation, decreases the glycolytic rate and thereby reduces ECM production and lung fibrosis. Here we provide intriguing evidence that increased FA availability, either pharmacologically (with metformin or rosiglitazone), genetically (*Angptl4* knockout), or by provision of FA in vitro, exerts anti-fibrotic effects. The key mechanism appears to be a metabolic shift in fibroblasts away from glycolysis towards greater mitochondrial fatty acid oxidation. This metabolic shift reduces collagen (ECM) production, mitigates TGFβ1-induced mitochondrial dysfunction, and lowers lactate production. Notably, these metabolic changes are associated with a downregulation of PDGFRα protein, implicating a potential link between FA metabolism and the function or abundance of PDGFRα-positive fibroblast population. Importantly, αSMA, a marker of myofibroblast activation, was only minimally affected, suggesting that modulation of fatty acid oxidation mainly affects specific fibroblast subtypes or functions. Overall, these results indicate that enhancing fatty acid oxidation represents a promising metabolic target to limit fibroblast-driven collagen deposition and the progression of pulmonary fibrosis.

## Materials and methods

### Human lung tissue

Lung specimens were obtained from idiopathic pulmonary fibrosis (IPF) patients who underwent lung transplantation at the Department of Surgery, Division of Thoracic Surgery, Medical University of Vienna, Austria. Written informed consent was obtained prior to lung transplantation. The study protocol and use of human tissue were approved by the institutional ethics committee (EK 976/2010 and EK 1417/2022, EK 1291/2025) of the Medical University of Vienna. Samples from downsized non-tumorous, non-transplanted donor lungs served as controls. All experiments involving human material were performed in accordance with the relevant guidelines and regulations.

### Experimental animals

*Angptl4* knockout mice on a C57Bl/6 background were obtained from Eli Lilly [37], while wild-type (WT) were generated in house by backcrossing to C57Bl/6 mice. *Pdgfra-CreERT2;tdTomato-flox* were used for metformin/vehicle treatment and *Acta2-CreERT2;fTom* mice were used for rosiglitazone/vehicle treatment. *Acta2-CreERT2;fTom* and *Pdgfra-CreERT2;tdTomato-flox* mice were induced with tamoxifen as previously described [14]. All animals were kept under conventional conditions on a chow-fed diet. Male mice were used for all experiments which were approved by the local authorities (Austrian Ministry of Education, Science and Culture, Austria) and performed in accordance with the EU directive 2010/63/EU. Animals were handled in parallel under identical conditions to minimize potential bias. For data acquisition and analysis, investigators were blinded to the experimental groups.

### Animal treatment

Metformin administration to *Pdgfra-CreERT2;tdTomato-flox* animals started one day after bleomycin treatment. Metformin was given in drinking water at the concentration of 0.3 mg/g of body weight (BW) - calculated based on an assumed daily water intake of 2 ml per 10 g body weight - for two weeks, after which lungs were harvested. For *Acta2-CreERT2;fTom* mice rosiglitazone administration started one day after bleomycin instillation, and it was given i.p. at the concentration of 10 mg/Kg every day for 2 weeks when lungs were harvested. Mice bleomycin treatment was administered as previously described [38]. Shortly, male *Angptl4* KO and WT counterpart, *Pdgfra-CreERT2;tdTomato-flox* and *Acta2-CreERT2;fTom* mice were anesthetized and an intra-tracheal administration of bleomycin (0.8 U/Kg BW for metformin treated mice, 1U/Kg BW for rosiglitazone treated mice and 1.5 U/kg BW for *Angptl4* KO and WT mice) (Sigma Aldrich, Austria) or saline solution (0.9% w/v NaCl) was applied with a MicroSprayer^®^ Aerosolizer (Penn-Century. Inc, USA) under light (~ 2%) isoflurane anesthesia. Mice were closely monitored till they completely recovered from anesthesia. At experimental endpoints, mouse lungs were perfused with PBS followed by 24 hours’ fixation with formalin and embedding with paraffin. The lungs from metformin and rosiglitazone treatment were perfused with PBS, inflated with optimal cutting temperature compound (Tissue Tek, USA), fixed overnight in 1% paraformaldehyde followed by overnight dehydration in 30% sucrose and stored at -80 °C.

### Lung function measurement

Respiratory mechanics were analyzed using the FlexiVent system (SCIREQ, France). Mice were anesthetized by intraperitoneal injection of ketamine (150 mg/Kg) and xylazine (20 mg/Kg). A tracheotomy was performed, and mice were connected to the FlexiVent system to perform measurement of the respiratory mechanics. Mice were ventilated at a rate of 150 breaths/min with a positive end-expiratory pressure (PEEP) of 3 cmH2O. After stable ventilation was achieved, three cycles of deep inflation, snapshot, prime-wave (QuickPrime-3) and pressure-volume loop (PVs-P) perturbations were applied for each mouse. All perturbations were automatically adjusted to the body weight of the mouse by the Flexiware software 7.6. Three measurements were averaged to give the represented value of one biological sample. After measurement, the mice were euthanized and the blood, broncho-alveolar lavage fluid (BALF) and lungs of all animals were collected for molecular and histological analysis.

### Non-esterified fatty acids (NEFA) measurement

Plasma concentrations of NEFAs were measured from *Angptl4* KO (*n* = 6–7) and WT (*n* = 6) counterpart according to the manufacturer’s instruction (FUJIFILM Wako Chemicals, Germany). In short, blood serum was isolated from the experimental animals. Working solutions of color reagents were added to freshly prepared standards and samples and incubated for 5 min at 37 °C. Data were acquired using the spectrophotometer CLARIOstar spectrophotometric plate reader (BMG Labtech, Germany) at a wavelength of 550 nm.

### Human parenchymal fibroblasts (hPF)

Human parenchymal fibroblasts (hPF) were isolated from donor and IPF parenchymal lung tissue following the laboratory protocol. Briefly, the tissue was finely minced using scissors and centrifuged in basic cell culture media (DMEM F12, Thermo Fisher Scientific, USA). The pellet was resuspended in complete medium (DMEM F12 with 10% FBS, 1% glutamine, 0,1% PenStrep, Dulbecco; Thermo Fisher Scientific, USA) and seeded into T75 cells culture flasks, where the cells were allowed to grow for 2–3 days. When the flask reached confluence, the cells were frozen in freezing media (complete medium + 10% DMSO) and stored in liquid nitrogen. For all experiments, hPF from passage 1–6 were used.

### Fatty acid treatment

Oleic acid (18:1), stearic acid (18:0) and palmitic acid (16:0) were prepared in combination with bovine serum albumin (BSA) (Sigma-Aldrich, Austria) in a ratio of 4:1 (FA: BSA) in a total concentration of 50µM, 85µM, 125µM and 250µM according to the Seahorse Bioscience protocol (North Billerica, USA). Before FAs treatment, cells were grown in low glucose medium (DMEM with 5% FBS, 1 g/L Glucose, 1% PenStrep, Thermo Fisher Scientific, USA).

### Cell growth analysis

For cell growth analysis we used a Nikon Ti2-U microscope and monitored cell growth by imaging cells every hour for 24 h. To assess the optimal dosages of the FA treatments, hPFs were treated with three FAs (18:1, 16:00 and 18:0) in two different concentrations (250 µM and 125 µM) added to the cells in cell culture medium (DMEM, Thermo Fisher Scientific, USA). After treatment the cells were grown and imaged for 48 h. The Etomoxir (ETO) dosage was selected based on literature reports [39] and its dose-dependent effect on OCR. To ensure that cell growth was not affected in the presence of 4µM etomoxir (ETO), hPFs were treated with ETO or vehicle control and their growth was monitored for 24 h. Images of treated and control wells were analyzed by the Fiji software [40] and the growth rate were assessed. A concentration of 125 µM for each FA (18:1, 16:0 and 18:0) was chosen based on the growth curve experiments and applied to hPF. BSA was used as a vehicle control. Cells were provided with fresh media containing the respective stimuli after 48 h and collected or fixed after a total of 72 h stimulation for immunoblotting and immunofluorescence, respectively.

### Lactate dehydrogenase (LDH) assay

Lactate dehydrogenase (LDH) (Roche, Austria) assay was used to detect cytotoxicity. Following the manufacturer’s protocol hPFs (10,000 cells/well) were plated directly in 12-well cell culture plates in serum-free medium (DMEM, 1 g/L Glucose, 1% PenStrep, Dulbecco, Thermo Fisher Scientific, USA). Each of the experimental wells was set up in triplicate. After 72 h of stimulation with the FA mix or control, with or without TGFβ, the medium was collected. Each sample was mixed with freshly prepared working reagent. After 30 min incubation, LDH concentrations were measured using the CLARIOstar plate reader (BMG LABTECH, Germany) at a wavelength of 492 nm. LDH concentration was calculated by subtracting the blank value (media-only control) from the sample values. These results were then divided by the automatically determined slope. Measurements were performed in triplicate. Data was analyzed using Microsoft Excel (Microsoft 365, version 2410, USA).

### Etomoxir (ETO) cell treatments

hPFs from donors and IPF patients were treated with an FA mix (125 µM for each FA − 18:1, 16:00 and 18:0) in presence or absence of TGFβ1 (10 ng/ml, Proteintech) for 48 h. The medium was then replaced with a FA-free medium, still containing TGFβ1/vehicle and ETO/vehicle (4µM) for an additional 24 h. Cells were subsequently harvested for protein and RNA isolation.

### Seahorse assay

Isolated hPF (50,000 cells/well) were plated directly into Seahorse XF96 cell culture microplates (Agilent Technologies, USA) and allowed to reach confluency. After 72 h stimulation with the free FA mixture the medium was replaced by Standard Oxidation Stress Test assay medium (10 mM glucose, 1 mM pyruvate, and 2 mM glutamine) and plates were incubated in a non-CO2 incubator for 60 min prior to the assay. Oxygen consumption rate (OCR) and extracellular acidification rate (ECAR) were measured in a Seahorse XFe96 extracellular flux analyzer (Agilent Technologies, USA) using XF Substrate Oxidation Stress Test Kits (XF Long Chain Fatty Acid Oxidation Stress Test Kit, XF Glucose/Pyruvate Oxidation Stress Test Kit) with and without etomoxir (ETO 4µM). First, basal respiration was established, then ATP production was blocked by oligomycin (1.5 µM). Basal respiration was taken as the last rate measurement before oligomycin injection subtracted by the minimum rate after Rotenone/Antimycin A and ATP production was calculated by subtracting the minimum rate measurement after oligomycin injection to the last rate measurement before oligomycin injection. For the etomoxir experiment 4 different donor hPF were mixed together and seed in equal amount per each well, prior starting stimulation. This experimental set up has been repeated 3 times. Etomoxir (4µM) or medium was added acutely after four measurement points for basal OCR and recorded for 8 measurements. Basal respiration and ATP production were calculated as last measurement point of Etomoxir/medium subtracted by the residual respiration or by the last oligomycin measurement respectively. Fatty acid oxidation dependence was calculated following the formula: Basal OCR-OCR (last ETO measurement) / ((Basal OCR (before ETO)-OCR (after Rotenone/Antimycin A)) *100 as reported by the manufacturer protocol. Measurements were performed in 5–10 technical replicates, rate measurement values from each well were normalized to their corresponding protein amounts. Data were analyzed using the Wave Software (Agilent Technologies, Version 2.6.1, USA).

### Lactate measurement

Isolated hPF (5,000 cells/well) were plated directly in 12 well cell culture plates in serum free media (DMEM, 1 g/L Glucose, 1% PenStrep, Dulbecco, Thermo Fisher Scientific, USA). After 72 h stimulation with the FA mixture the medium is collected and subjected to the purchased kit for lactate measurement (EnzyChrom™ Glycolysis Assay Kit, BioAssay Systems, United States). Following the manufacturer protocol, standards were prepared using the collected cell media. Each sample was mixed with the freshly prepared Working reagent. After 30-minute incubation lactate concentration were measured using the CLARIOstar (BMG LABTECH, Germany) at 565 nm. Lactate concentration was calculated by subtracting the blank value (only media standard value) from the sample values. Those results were then divided by the automatically determined slope. Measurements were performed in duplicates. Data was analyzed using the Microsoft Excel (Microsoft 365, version 2410, USA).

### Western blot

Lung tissue from all experimental mice was homogenized with the homogenizer Magna Lyser in RIPA buffer (Santa Cruz, USA) supplemented with protease and phosphatase inhibitors (ThermoFisher Scientific, USA). Cells were collected using the Laemmli buffer prepared in house. In short, 1,5% SDS and 1,5 M Tris-CL pH 6.8 were added to cells previously washed in PBS (Thermo Fisher Scientific, USA). The collected lysate was supplemented with 10% of β-mercaptoethanol with glycerol and bromophenol blue. Proteins were separated using the 8–12% gels-depending on the size of the detected protein and SDS PAGE electrophoresis, wet transferred on polyvinylidene fluoride (PVDF) membranes (ThermoFisher Scientific, USA). The following antibodies were used: CPT1α (Cat. No: #12252, Cell Signaling, Netherlands), COL1A1 (Cat. No: 1310, Southern Biotech, USA), PDGFRα (Cat. No: #3174, Cell Signaling, Netherlands), αSMA (Cat. No: MA5-11547, Thermo Fisher Scientific Technology, USA), pro-SPC (Cat. No: AB3786, Millipore, USA), pAMPK (Cat. No: #2535, Cell Signaling, Netherlands), AMPK (Cat. No: #5831, Cell Signaling, Netherlands), BIP (Cat. No: #3177, Cell Signaling, Netherlands) and α-tubulin (Cat. No: #2144, Cell Signaling, Netherlands) was used as loading control.

### RNA isolation and qPCR

Total RNA was isolated using the inuPREP RNA isolation kit (Analytik Jena, Germany) for cells, and the Qiagen RNA isolation kit for mouse lung tissue according to manufacturer’s instructions. After assessing the RNA concentration, cDNA synthesis was performed by using iScript kit (BioRad, USA) or the qScriptTM cDNA Synthesis kit (QuantaBio, VWR, Austria) according to manufacturer’s instructions. Quantitative real time polymerase chain reaction (qRT-PCR) was performed using the BlueS’Green qPCR kit (Biozym, Germany) on LightCycler 480 (Roche Applied Science, Germany). The threshold cycle difference (ΔCT) was calculated the equation ΔCT = CT housekeeping gene – CT gene of interest)) Beta-2-microglobulin (β2M) or porphobilinogen deaminase (PBGD) or were use as housekeeping genes. The primer list is given in Supplementary Table 1.

### Immunofluorescence staining

Paraffin sections and cryosections were prepared as previously described [14, 41]. In short, paraffin sections after de-paraffinization and re-hydration were treated with antigen retrieval solution (DAKO solution pH 6/9; Agilent Pathology Solution, Germany) while cryosections were fixed with 4% paraformaldehyde (Thermo Fisher Scientific, USA). Both paraffin sections and cryosection were blocked in 3% BSA in PBS (Thermo Fisher Scientific, USA), 2.5% Normal Horse Serum Blocking Solution (Vector Laboratories, USA) and 5% Donkey Serum in PBS (Jackson ImmunoResearch, United Kingdom). Cells in chamber slides were firstly fixed with 4% paraformaldehyde (Thermo Fisher Scientific, USA), permeabilized in 0.2% Triton (ThermoFischer Scientific, USA) and blocked the same way as described above. Following antibodies were used: Alpha SMA (1:500, Cat No: CL674-67735, Omni Life Science, Switzerland), SMA Cy3 (1:100, Cat No: C6198, Sigma), PDGFRα (1:500, Cat. No: ab124392, Abcam, USA for cell staining and 1:1000 Cat. No: #3174, Cell Signaling, Netherlands for lung tissue staining), COL1A1 (1:500, Cat. No: 1310, Southern Biotech, USA). For the lipid staining BODIPY 493/503 (1:200, 4,4-Difluoro-1,3,5,7,8-Pentamethyl-4-Bora-3a,4a-Diaza-s-Indacene, Thermo Fisher Scientific, USA) was used. PDGFRα signal was enhanced by Tyramide Signal Amplification Kit (Thermo Fischer Scientific, USA) according to manufacturer’s instruction. All antibodies were diluted in 3% BSA in PBS. Secondary antibodies were conjugated with Alexa Fluor 488, 555 or 647 (1:500, all from Thermo Fischer Scientific, USA) were used and nuclear staining was done by DAPI containing mounting media (Vector Laboratories, USA). Sections and chambers slides were imagined with the Leica SP8 clsm-microscope (TCS SP8 X, Leica, Austria) using an 63x objective (HC PL APO 63x/1.40 OIL CS2, 1.40 Numerical aperture, Leica, Austria) or on the Nikon A1 microscope (Nikon, Austria) in 20× objective (CFI Plan Apo λ 20x/0.75 numerical aperture, Nikon, Austria), 40x objective (CFI Plan Apo λ 40x/0.95 numerical aperture, Nikon, Austria) and 60x objective (CFI Plan Apo λ 60x Oil/1.40 numerical aperture, Nikon, Austria). Approximately 3–10 pictures were acquired for each tissue section and images were processed by the FIJI (ImageJ, version 1.51 h, LOCI, USA). Quantification of the mean fluorescence intensity was performed using QuPath (version 0.5.1). For immunofluorescence images acquired from chamber slide–cultured cells, nuclei were first detected using the DAPI channel. To approximate the cytoplasmic region, a cell expansion of 5 µM from the nuclear detection area was set and this enlarged region was defined as the cytoplasmic compartment for subsequent fluorescence intensity. For quantification of the average lipid droplet volume with Bodipy staining, 10 z-stacks were acquired from each section and then calculated using the Kapur–Sahoo–Wong (maximum entropy) method as previously reported [42] and average lipid droplet volume per cell for each image was calculated based on the nuclei counts.

### Histological staining

Sirius Red staining and Masson’s Trichrome were performed according to the manufacturer’s instruction (Thermo Fisher Scientific). Both paraffin and cryosections were used. To ensure unbiased quantification, the operator was blinded to group allocation, and mice were coded with anonymized identifiers., Analysis was performed with the Visiopharm software (Visiopharm, Denmark). The percentage of collagen area (Sirius Red and Blue area in Masson´s Trichrome area) was quantified and normalized to the tissue area of the analyzed lung.

### scRNA-Seq data analysis

To investigate gene expression in human IPF/donor samples and bleomycin/saline mice model, we utilized previously published scRNA datasets (GSE135893, GSE201698) [43], https://www.ncbi.nlm.nih.gov/geo/query/acc.cgi?acc=GSE201698]. For GSE135893, dataset was downloaded, normalized, batch corrected, integrated, and subjected to dimensionality reduction and clustering, as described in the original publication. We performed normalization with default parameters and dimensionality reduction with selected parameters: 20 components, 30 neighbors, distance of 0.4. We subset the specific fibroblasts populations: Fibroblasts, Myofibroblasts and PLIN2 + Fibroblasts from the controls and IPF samples. For GSE201698, quality control removed cells with RNA count and RNA features ≥ 3 times of mean absolute deviation (MAD). Each sample was normalized individually and integrated using default Seurat integration [44]. Integrated data was scaled with regressing out the mitochondrial gene percentage and cell cycle scores. PCA and UMAP were performed using 10 dimensions. Clustering was determined using a resolution of 0.3. Fibroblasts and SMC clusters were identified by calculating population markers scores [45]. Gene visualizations for all scRNA datasets were done using built-in visualizations functions (FeaturePlot, DimPlot, VlnPlot) in Seurat. Dot plots were visualized with ggplot2 package (Wickham H. ggplot2: Elegant Graphics for Data Analysis. Springer-Verlag New York, 2016. ISBN: 978-3-319-24277-4, https://ggplot2.tidyverse.org). Computational analyses were performed in Seurat version 3.2 [43] in the R environment (RStudio Team (2020). RStudio: Integrated Development for R. RStudio, PBC, Boston, 2020. MA URL http://www.rstudio.com/).

### Bulk-RNA seq analysis

To understand the genetic expression of metabolic related genes in different populations of fibroblasts in bleomycin treated mice, we use our previously published data set (GSE126205, https://www.ncbi.nlm.nih.gov/geo/query/acc.cgi?acc=GSE126205) [14]. This data set contains the data from Pdgfra-CreERT2;tdTomato-flox and Acta2-CreERT2;fTom bleomycin treated mice, sacrificed at day 14, and was pre-analyzed and normalized as described previously [14]. Genes were ranked by significance (p-value < 0.05) and fold change (absolute fold change > 1). Visualizations including volcano plots, dot plots, and Venn diagrams were performed on RStudio [46] using ggplot2 package [47] and tidyverse package [48]. HeatMap was generated using pheatmap package [49]. Gene enrichment analysis was performed using the Enrichr platform, using WikiPathways 2024 Mouse, GO Biological Process 2023 and GO Molecular Function 2023 as databases (the Ma’ayan Lab, https://maayanlab.cloud/Enrichr/) [50]. Only significant pathways (p-values < 0.05) were retained for further analysis.

### Statistical analysis

Statistical analysis was performed with GraphPad Prism 10 (Version 10.6.0 (890), GraphPad Software, USA). Data are presented as single data point with standard error of mean (SEM), except for paired cells-derived results which are represented as single data point line-connected due to pairwise comparison. For comparison of two groups, t-test was carried out. Differences between more than two groups were assessed by one-way ANOVA followed by Šidák or Dunett’s multiple comparison correction. Specifically, Šidák correction was performed when specific pair comparisons were carried out, while Dunnet’s correction was performed when multiple treatment groups were compared to a single control group. Growth curves (with FA or with etomoxir) where analyzed with two-way ANOVA. Significance is indicated by an asterisk on the graphs. A p value < 0.05 was considered significant for all analysis. All experiments were designed with matched control conditions to enable statistical comparison.

### Data availability

The datasets generated analyzed during the current study are available in the NCBI repository under corresponding links: GSE135893 (https://www.ncbi.nlm.nih.gov/geo/query/acc.cgi?acc=GSE135893), GSE201698 (https://www.ncbi.nlm.nih.gov/geo/query/acc.cgi?acc=GSE201698), GSE126205 (https://www.ncbi.nlm.nih.gov/geo/query/acc.cgi?acc=GSE126205).

Research involving animals, their data or biological material were approved by Austrian Ministry of Education, Science and Culture, Austria and performed in accordance with the EU directive 2010/63/EU.

Research involving human participants, their data or biological material performed in line with the principles of the Declaration of Helsinki. Approval was granted by the institutional ethics committee (EK 976/2010 and EK 1417/2022, EK 1291/2025) of the Medical University of Vienna. Consent was obtained before lung transplantation. Lungs specimens were obtained from idiopathic pulmonary fibrosis (IPF) patients who underwent lung transplantation at the Department of Surgery, Division of Thoracic Surgery, Medical University of Vienna, Austria. Figures were designed in the CorelDRAW^®^ Graphics Suite 2024.

## Results

### Fibroblasts exhibit enhanced glycolysis and suppressed fatty acid metabolism during bleomycin-induced fibrosis

**Fig. 1:**
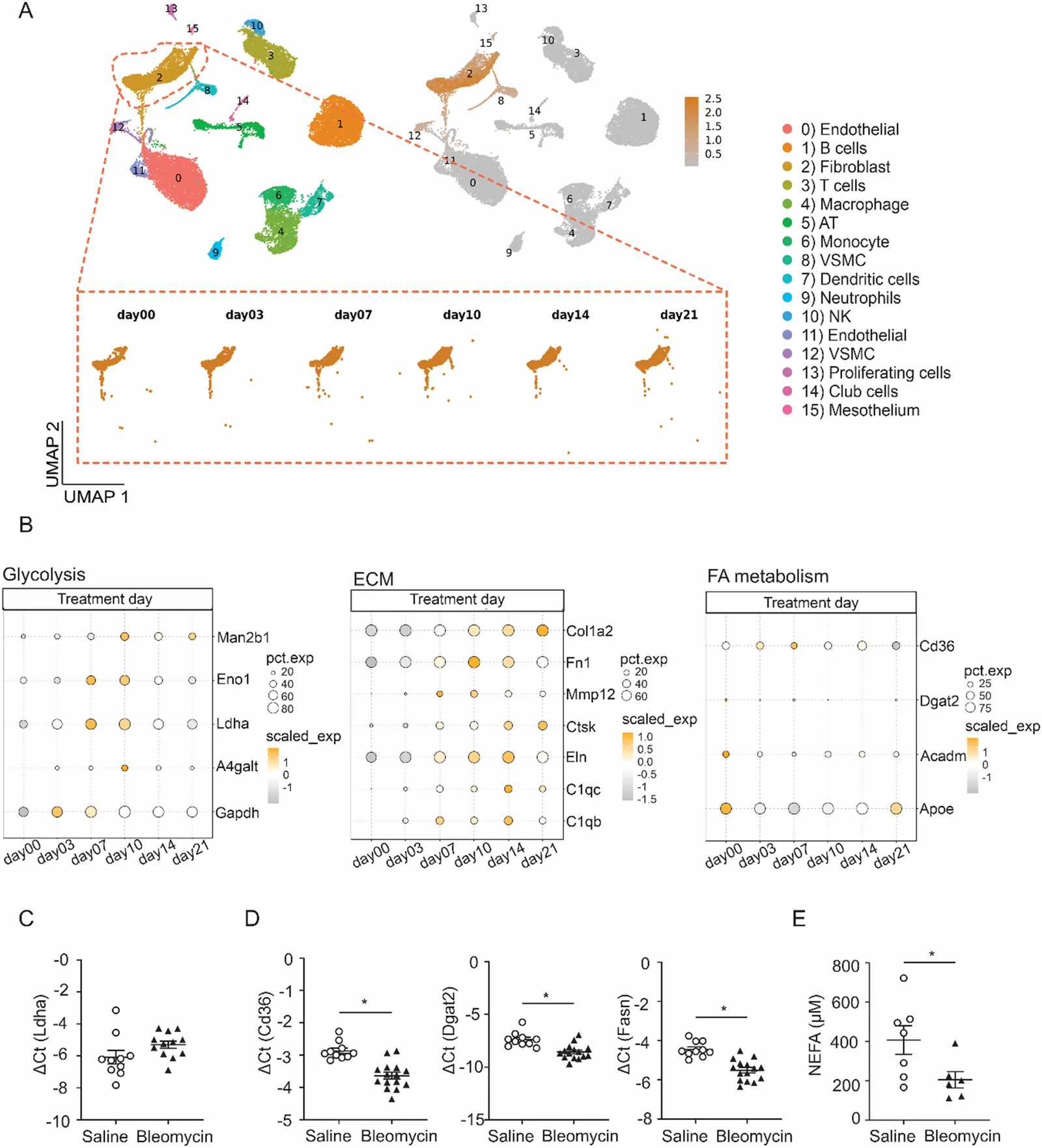
Bleomycin-induced fibrosis activates glycolysis and suppresses fatty acid metabolism. (**A**) Uniform manifold approximation and projection (UMAP) of the mouse scRNA-Seq data (GSE201698) from lung homogenate of bleomycin treated WT mice at day 0, 3, 7, 10, 14, and 21. Colour represents different cell populations, the inset shows the fibroblasts population at the investigated time points. (**B**) Dot plots of the most upregulated genes associated with glycolysis, extracellular matrix (ECM) and fatty acid (FA) metabolism at the reported time points in the selected fibroblast population. Gene expression analysis of (**C**) *Ldha*; (**D**) *Cd36*, *Dgat2* and *Fasn* performed in lung homogenates of WT animals treated with bleomycin or saline and sacrificed at day 14 (*n* = 10–15). (**E**) Non-esterified fatty acids (NEFA) measurement in the serum of saline and bleomycin treated WT mice 14 days post-administration (*n* = 6–7). Data are presented as mean ± SEM. Statistical significance was determined using an unpaired two-tailed Student’s t-test. *P* < 0.05 were considered statistically significant

**Fig. 2:**
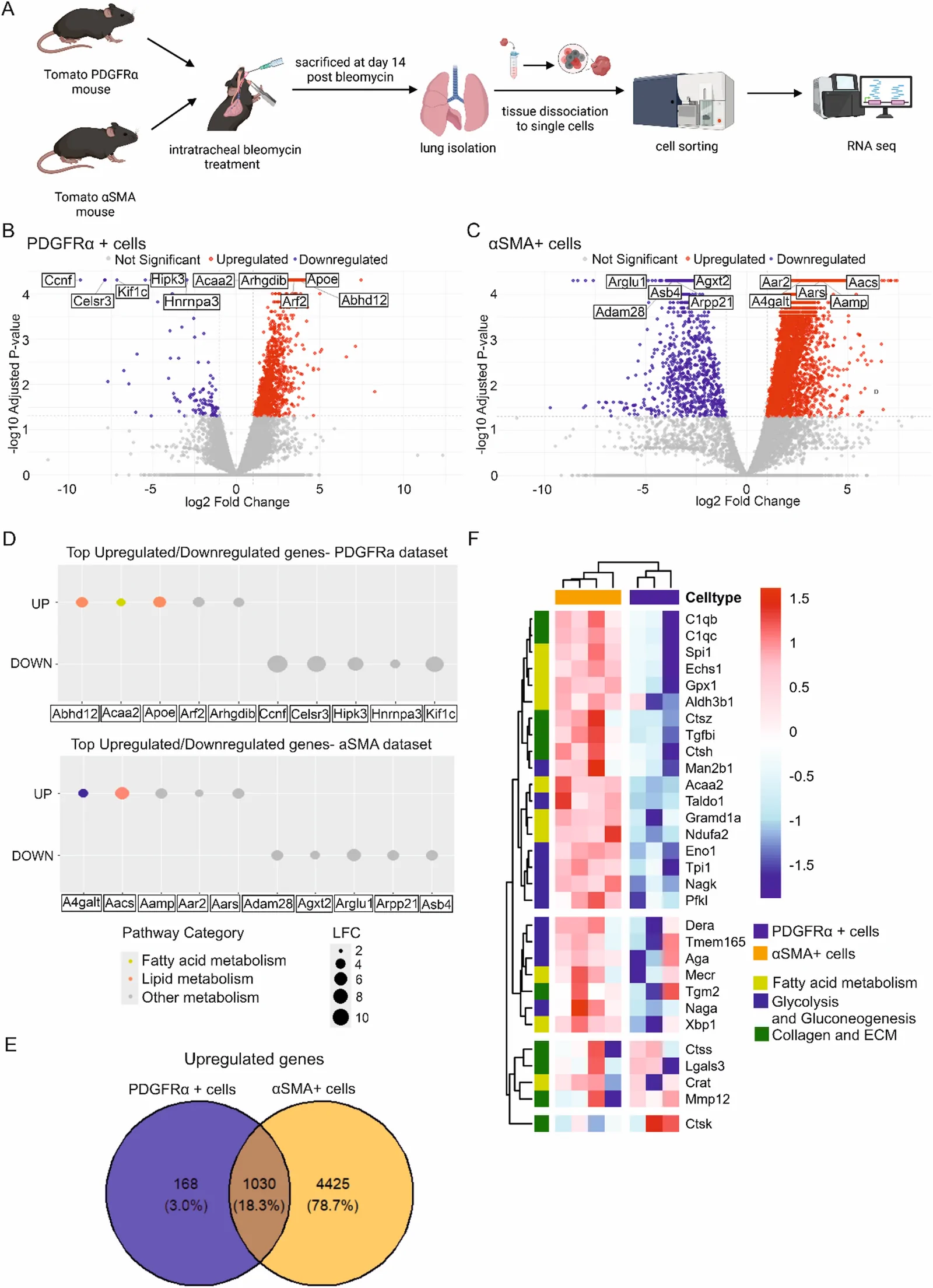
Bleomycin-induced fibrosis strongly influences the metabolic profile of Pdgfrα and αSma positive cells. (**A**) Graphical representation of the experimental workflow. Volcano plots of (**B**) *Pdgfrα* (*n* = 3) and (**C**) *αSma* (*n* = 4) positive cells highlighting the 5 most upregulated and downregulated genes in the datasets. (**D**) Dot plots highlighting the most upregulated and downregulated genes in both datasets. (**E**) Venn diagram showing the common upregulated genes between *Pdgfrα+* and *αSma+* cells. (**F**) Heatmap displaying the 10 most upregulated genes associated with fatty acid metabolism, glycolysis and ECM pathways in *Pdgfrα +* and *αSma+* cells following Enrichr analysis

To assess which metabolic pathways are preferentially active during bleomycin induced lung fibrosis, we analyzed a publicly available dataset. Genes involved in glycolysis, FA metabolism and extracellular matrix remodeling were analyzed in fibroblast populations of bleomycin treatment time course (Fig. 1A and B). Genes involved in glycolysis were upregulated in the early phases of bleomycin (day 7–10) followed by strong increase in ECM genes (day 10–21). Interestingly, genes involved in FA metabolism appeared to be more highly expressed in saline-treated mice compared to bleomycin treatment (e.g. *ApoE*, *Acadm2*, *Dgat2*) (Fig. 1B). To corroborate these results, we performed gene expression analysis on 14 days bleomycin treated WT mice lungs compared to their saline counterpart. We observed a slight increased expression of lactate dehydrogenase A (*LdhA*) (Fig. 1C), suggesting an increase in lactate production from the glycolytic flux, decrease of genes involved in FA transport (*Cd36*), FA storage (*Dgat2*) and FA synthesis (*Fasn*) (Fig. 1D) together with a decrease of master regulator of lipid metabolism (*Srebf1*) and a tendentially lower *Acsl1* suggesting lower FA oxidation (Supplementary Figure 1A-B). In line with the expression level of these genes, we observed a significant decrease of circulating FA in serum from bleomycin treated WT mice compared to saline counterpart (Fig. 1E). We have previously shown that two population of fibroblasts, labelled as *αSma+* and *Pdgfrα+* cells, with minimal transdifferentiating potential, similarly contribute to collagen production in both human IPF and in bleomycin-induced fibrosis [14]. To determine whether the glycolytic switch and reduced fatty acid metabolism represent a common feature of both fibroblast types or are specific to one population, we analyzed bulk RNA-seq datasets derived from mice treated with saline or bleomycin for 14 days, in which fibroblasts were sorted into *αSma+*⁺ and *Pdgfrα+* populations (Fig. 2A). Volcano plots show the differentially upregulated and downregulated genes in both *αSma+* and *Pdgfrα +* cells, suggesting a higher number of differentially expressed genes in the *αSma+* dataset, compared to the *Pdgfrα+* dataset (Fig. 2B-C). Interestingly, from the five most upregulated genes in both datasets, 3/5 and 2/5 genes respectively belonged to metabolic pathway in *Pdgfrα+* and *αSMA+* datasets (Fig. 2D). Specifically, in *Pdgfrα+* cells a lipase, (lysophosphatidylserine lipase- *Abdh12*), a key enzyme in fatty acid oxidation (Acetyl-CoA Acyltransferase 2 - *Acaa2*) and a protein involved in lipid transport (Apolipoprotein E -*ApoE*) were among the 5 most upregulated genes (Fig. 2D). In *αSma+* cells, a glucosyl transferase (1,4-galactosyltransferase - *A4galt*) and an enzyme important for lipid synthesis (acetoacetyl-CoA synthetase - *Aacs*) were two of the five most upregulated genes (Fig. 2D). To understand the commonalities of the two fibroblast populations, Venn diagram analysis was performed (Fig. 2E). Comparison of both data sets showed 1030 common upregulated genes (Fig. 2E) and 16 downregulated genes (Supplementary Fig. 2A) between the *Pdgfrα+* and *αSma+* cells. To reveal whether the common genes were linked to metabolic pathways or to ECM production, the upregulated genes were analyzed by Enricher analysis tool. As depicted in Fig. 2F, although both fibroblasts population display changes in metabolic and in ECM related genes, *αSma+* cells appear to display not only higher upregulation of metabolic genes compared to *Pdgfrα+* cells but also of ECM related genes (Fig. 2F), potentially suggesting a stronger metabolic and ECM effect of bleomycin on *αSma+* cells compared to *Pdgfrα+* cells. Altogether these results suggest that, although there are intrinsic differences in *αSma+* and *Pdgfrα+* cells, metabolic alteration is a predominant component of bleomycin induced changes in both fibroblast subpopulations.

### Metabolic interventions attenuate fibrosis and increase CPT1 expression

**Fig. 3:**
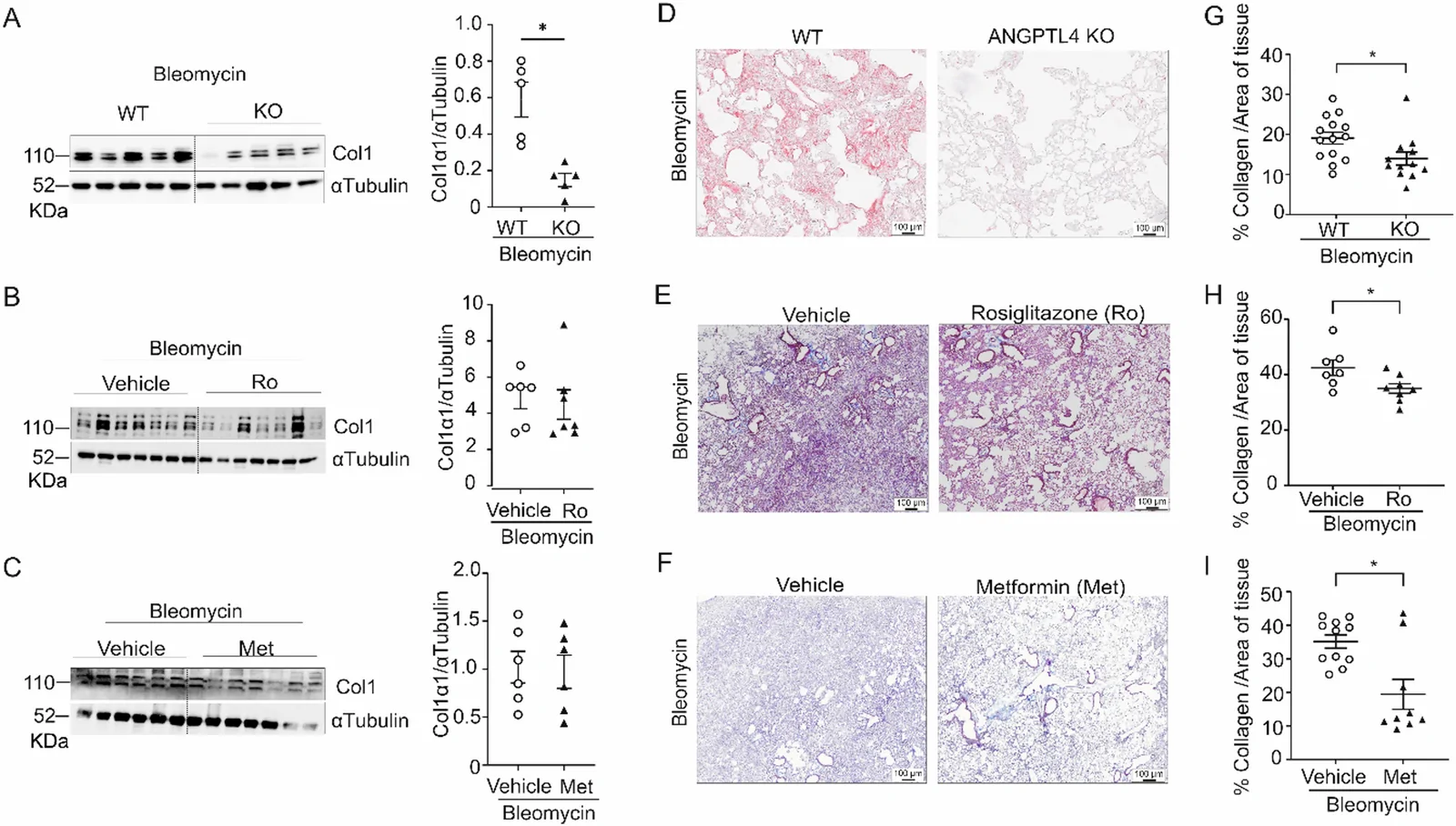
Metabolic interventions decrease bleomycin/induced collagen deposition. Western blot analysis of Collagen-I (Col1) and its relative quantification in (**A**) *Angptl4* KO and WT mice (n = 5 per group), (**B**) rosiglitazone (Ro) (n = 7 per group) and (**C**) metformin (Met) (n = 7 per group) treated mice subjected to bleomycin and sacrificed at day 14 post-administration. Protein levels were quantified relative to the loading control α-tubulin. Representative collagen staining and relative quantification in (**D**, **G**) *Angptl4* KO and WT mice using Sirius Red (n = 12–15 per group), (**E**, **H**) rosiglitazone-treated mice (Ro) using Masson’s trichrome staining (n = 7–8 per group) and (**F**, **I**) metformin-treated mice (Met) using Masson’s trichrome staining (n = 9–11 per group), all subjected to bleomycin and sacrificed 14 days post-administration. Scale bar: 100 μm. Data are presented as mean ± SEM. Statistical significance was determined using an unpaired two-tailed Student’s t-test. *P* < 0.05 were considered statistically significant

In order to determine whether the previously reported anti fibrotic effect of rosiglitazone, metformin and the genetic ablation of *Angptl4* are mediated by a direct effect on metabolism of the two fibroblast subpopulations, we administered bleomycin to those models. Bleomycin treated mice were administered with either rosiglitazone or metformin and their respective vehicle. Similarly, *Angptl4* KO mice and the WT counterpart were subjected to bleomycin and saline. While the genetic ablation of *Angptl4 in vivo* led to a substantial decrease of bleomycin-induced collagen protein level compared to WT (Fig. 3A), no statistical difference at the protein level was detected in bleomycin mice subjected to rosiglitazone and metformin treatment compared to vehicle control (Fig. 3B-C). At the mRNA level, collagen was significantly reduced in the rosiglitazone treatment group compared to vehicle control, however no changes were observed in the metformin treated group (Supplementary Fig. 3A-B). Due to the high patchiness and heterogeneity of the bleomycin model [51, 52] and the possible bias resulting from the analysis of protein/mRNA obtained from lung homogenate derived from a smaller piece of tissue, we opted to analyze the whole left lobe lung by Sirius Red and Masson’s Trichrome staining. In line with previously reported studies [31–33], we observed a consistent decrease of collagen amount in all three investigated models as depicted by collagen quantification analysis (Fig. 3D-I). Lung compliance was significantly improved only in metformin treatment, while no changes were detected in the other investigated models (Supplementary Fig. 3C-E). Of note, no difference was observed for the epithelial marker pro-Spc (Supplementary Fig. 4A-C).

**Fig. 4:**
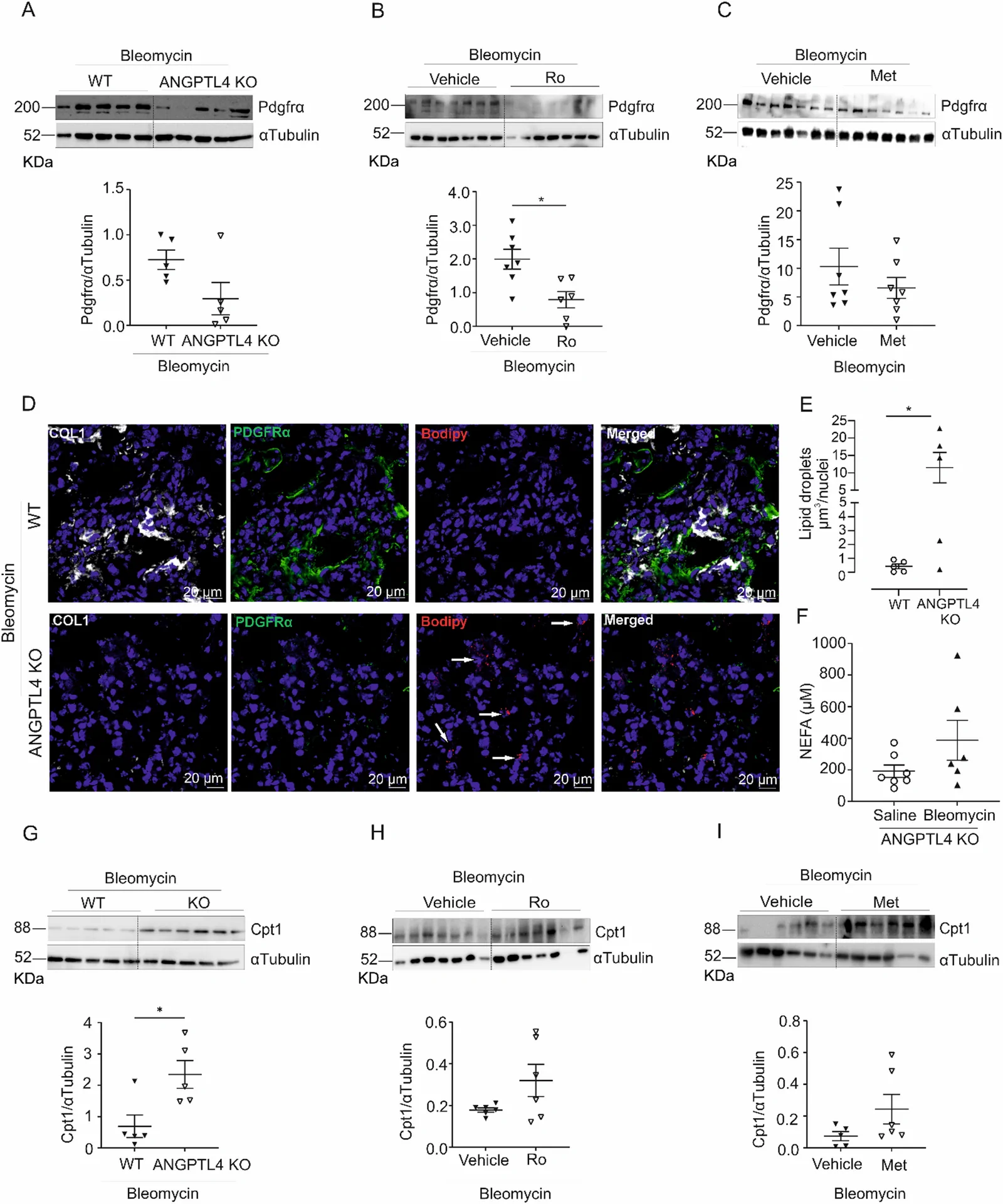
Metabolic interventions reduce Pdgfrα protein expression and enhance fatty acid metabolism. Western blots analysis and relative quantification of Pdgfrα in (**A**) *ANGPTL4* KO and WT mice (*n* = 5 per group). (**B**) rosiglitazone (Ro)-treated mice (*n* = 7 per group), and (**C**) metformin (Met)-treated mice (*n* = 7 per group) subjected to bleomycin administration and sacrificed 14 days post-treatment. (**D**) Representative images of triple immunofluorescence staining for COL1 (white), platelet-derived growth factor receptor α (Pdgfrα; green), and BODIPY (red) in lung tissue from *Angptl4* KO and WT mice 14 days after bleomycin administration. Scale bar: 20 μm (*n* = 4–5). (**E**) Quantification of lipid droplet volume normalized to the number of nuclei (µm³/nuclei). **(F**) Non-esterified fatty acid (NEFA) levels measured in the serum of saline- and bleomycin-treated mice 14 days post-administration (*n* = 6–7). Western blot analysis and relative quantification of Cpt1 protein expression in lung homogenates from (**G**) Angptl4 KO and WT mice (*n* = 5 per group), (**H**) rosiglitazone-treated mice (*n* = 6–7 per group), and (**I**) metformin-treated mice (*n* = 6 per group), all subjected to bleomycin administration and sacrificed 14 days post-treatment. Protein levels were normalized to the loading control α-tubulin. Data are presented as mean ± SEM. Statistical significance was determined using an unpaired two-tailed Student’s t-test. *P* < 0.05 were considered statistically significant

To delineate which fibroblast population was mostly influenced by the pharmacological treatment and genetic intervention, we checked the expression level of αSma and Pdgfrα. Although we observed in all three models a slight downward trend in αSma protein (Supplementary Fig. 4A-C), the Pdgfrα protein level was decreased in *Angptl4* KO mice compared to WT group and in the rosiglitazone treatment group. Additionally, downward trend was detected upon metformin treatment. Our results align with existing literature by showing a downward trend in αSma protein level following this metabolic intervention [33–35] more importantly, we extend these findings by demonstrating a robust in vivo reduction in Pdgfrα. (Fig. 4A-C). Moreover, we could corroborate the decreased Pdgfrα expression on protein level in *Angptl4* KO mice at the immunofluorescence level (Fig. 4D). This reduction in Pdgfrα was accompanied by a concomitant decrease in collagen, as shown by Col1 staining (Fig. 4D). In order to assess whether lack of Angptl4 might lead to decreased Pdgfrα and collagen amount by enhancing lipid metabolism, we performed Bodipy staining in WT and *Angptl4* KO mice subjected to bleomycin. As shown in Fig. 4D and quantified in Fig. 4E, lipid droplets were more frequent in bleomycin treated *Angptl4*4 KO mice compared to WT littermates (Fig. 4D red staining highlighted by the arrow). As the enhanced lipid accumulation in the lung could be a consequence of a higher mobilization of FA from the periphery, we assessed circulating FAs levels in *Angptl4* KO subjected to saline or bleomycin treatment. Indeed, contrary to bleomycin administration in WT mice where free FA were decreased (Fig. 1E), in *Angptl4* KO mice we observed that bleomycin tendentially enhanced circulating fatty acids compared to saline counterpart (Fig. 4F). Next, we investigated whether the reduced Pdgfrα and collagen protein levels were associated with enhanced fatty acid oxidation. Consistent with this possibility, Cpt1, a key enzyme regulating mitochondrial fatty acid oxidation, was increased across all three models, reaching significance in *Angptl4* KO and showing a trend toward an increase in rosiglitazone and metformin (Fig. 4G–I).

### Fatty acid availability enhances mitochondrial metabolism and suppresses TGFβ1-induced collagen production in donor fibroblasts

**Fig. 5:**
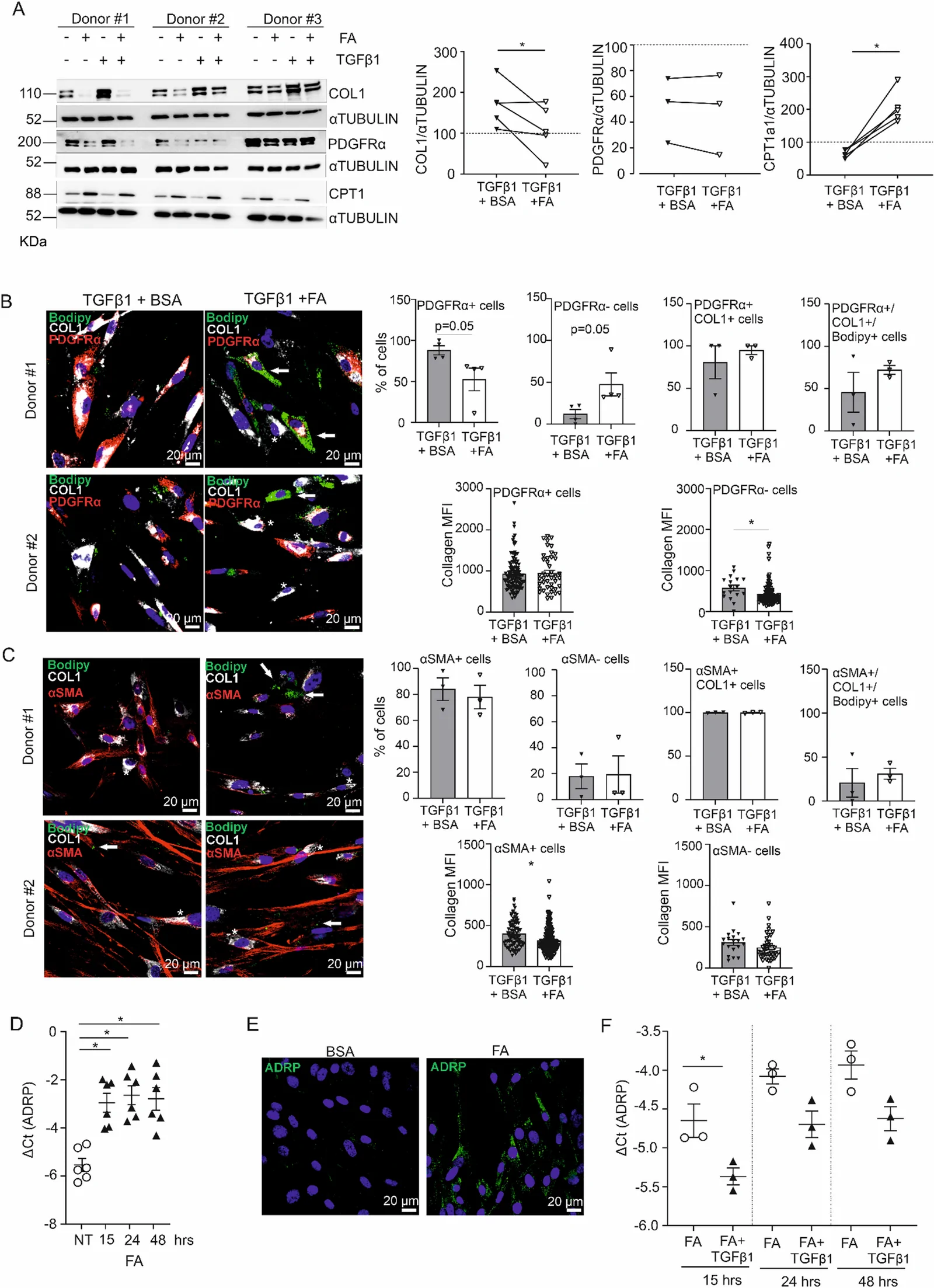
Fatty acid availability decreases TGFβ1-induced collagen production and enhances fatty acid oxidation. (**A**) Representative western blot and relative quantification of COL1, PDGFRα, and CPT1α in human pulmonary fibroblasts (hPFs) isolated from donor lungs and treated with a fatty acid (FA) mixture (16:0, 18:0, 18:1) for 72 h with or without TGFβ1 (10 ng/ml) (*n* = 5). Protein levels were normalized to the loading control α-tubulin. The dotted line indicates vehicle control (BSA-treated) cells. (**B–C**) Representative images of triple immunofluorescence staining showing (**B**) COL1 (white), PDGFRα (red), and BODIPY (green) (*n* = 4) and (**C**) COL1 (white), αSMA (red), and BODIPY (green) (*n* = 3) in hPFs treated with the FA mixture (16:0, 18:0, 18:1) for 72 h with or without TGFβ1 (10 ng/ml). Quantification of the percentage of positive cells and collagen mean fluorescence intensity (MFI) is shown in panels B and C. Scale bar: 20 μm. Arrows indicate lipid droplet–positive cells, and asterisks indicate collagen-positive cells. (**D**) Gene expression analysis of *ADRP* in hPFs treated with FA at different time points up to 48 h.(**E**) Representative immunofluorescence images of ADRP staining (green) in hPFs treated with the FA mixture (16:0, 18:0, 18:1) for 48 h (*n* = 3). Scale bar: 20 μm. (**F**) Gene expression analysis of *ADRP* in hPFs treated with the FA mixture (16:0, 18:0, 18:1) at 15 h, 24 h, and 48 h in the presence or absence of TGFβ1 (10 ng/ml) (*n* = 3). Data are presented as mean ± SEM. Statistical significance for panel A and F was determined using a paired two-tailed Student’s t-test, while panels B–C were analyzed using an unpaired two-tailed Student’s t-test. Panel D was analyzed using a repeated-measures one-way ANOVA followed by Dunnett’s multiple comparisons test. *P* < 0.05 was considered statistically significant

We hence aimed to prove whether enhancement of FA availability would be sufficient to decrease collagen production from human lung derived parenchymal fibroblasts (hPF). We used the three most common FA circulating in the serum of healthy adults [26] (oleic acid (18:1), palmitic acid (16:0) and stearic acid (18:0)) in a physiological concentration [53, 54] and assessed their influence on hPF growth. As shown in Supplementary Fig. 5, the two tested concentrations of FA mixture did not influence donor hPF growth. To minimize the risk of FA overload and to remain within a physiological range, we chose the lower of two tested concentrations (18:1/16:0/18:0; 125µM:125µM:125µM) to understand the effect of the FA mix on TGFβ induced collagen production. As shown in Fig. 5, the FA mixture decreased TGFβ1-induced collagen-I levels at both protein and mRNA level (Fig. 5A and Supplementary Fig. 6A, ). To test the dose dependency of FA mix on the collagen effect, we assessed COL1 and PDGFRA protein level upon 50 µM and 85 µM of each FA. Although the results did not reach statistical significance, we could still observe a decreasing trend in concomitantly FA and TGFβ1 treated samples compared to TGFβ1 stimulation (Supplementary Fig. 6B). However, contrary to the in vivo results, the FA mixture was not able to decrease PDGFRA protein level (Fig. 5A). The decrease of collagen-I induced by FA was accompanied by a sharp increase of CPT1 (Fig. 5A and Supplementary Fig. 6C). To ensure that the observed collagen effect was not a consequence of ER stress or cell toxicity, we evaluated the level the Binding Immunoglobulin Protein (BIiP), marker of endoplasmic reticulum stress [55] and lactate dehydrogenase (LDH) a marker of cell toxicity [56]. As shown in Supplementary Fig. 6D and E, both BIP and LDH level did not exceed the TGFβ1 induced levels s confirming lack of additive stress and toxicity of the used FA mix. Immunofluorescence analysis further confirmed the biochemical findings. FA treatment reduced collagen-I deposition and increased intracellular lipid droplet accumulation, as indicated by BODIPY staining (Fig. 5B–C). Quantification of cell populations revealed a decrease in the amount of PDGFRα+ cells in the FA + TGFβ1 condition compared with TGFβ1 stimulation alone, accompanied by an increase of PDGFRα- population. While collagen expression in PDGFRα+ cells remained unchanged, collagen mean fluorescence intensity wase reduced in PDGFRα-⁻ cells. Analysis of α-smooth muscle actin (αSMA) expression revealed no change in the proportion of αSMA+ cells between conditions. However, αSMA+ fibroblasts exhibited reduced collagen production following FA co-treatment compared with TGF-β1 stimulation alone. The abundance of double- (PDGFRα /COL1; αSMA/COL1) and triple-positive populations, including (PDGFRα+/COL1+/BODIPY+ and αSMA+/COL1+/BODIPY+ cells) remained unchanged. To delineate how enhanced lipid storage and enhanced β oxidation are co-existing in FA stimulated cells, we first analyzed ADRP as a marker of lipid droplets formation. FA treatment increased ADRP protein and mRNA expression as early as 15 h (Fig. 5D–F and Supplementary Fig. 7A), indicating active lipid droplet formation. In contrast, TGFβ1 stimulation reduced *ADRP* mRNA levels, suggesting increased utilization of stored lipids (Fig. 5F). Interestingly we observed that upon concomitant FA and TGFβ1stimulation, hPF enhanced expression of *DGAT1*, an enzyme involved in the formation of lipid droplets, after 24 h (Supplementary Fig. 7A), and downregulation at 48 h or no change were observed on expression level of hormone-sensitive lipase (*HSL*) and adipose triglyceride lipase (*ATGL*) respectively, enzymes involved in TG breakdown. Although this data might seem contradictory, it suggests that TGFβ1 supplementation induced breakdown of lipid droplet and usage of fatty acids, while maintaining the storage process active to protect for lipotoxicity.

**Fig. 6:**
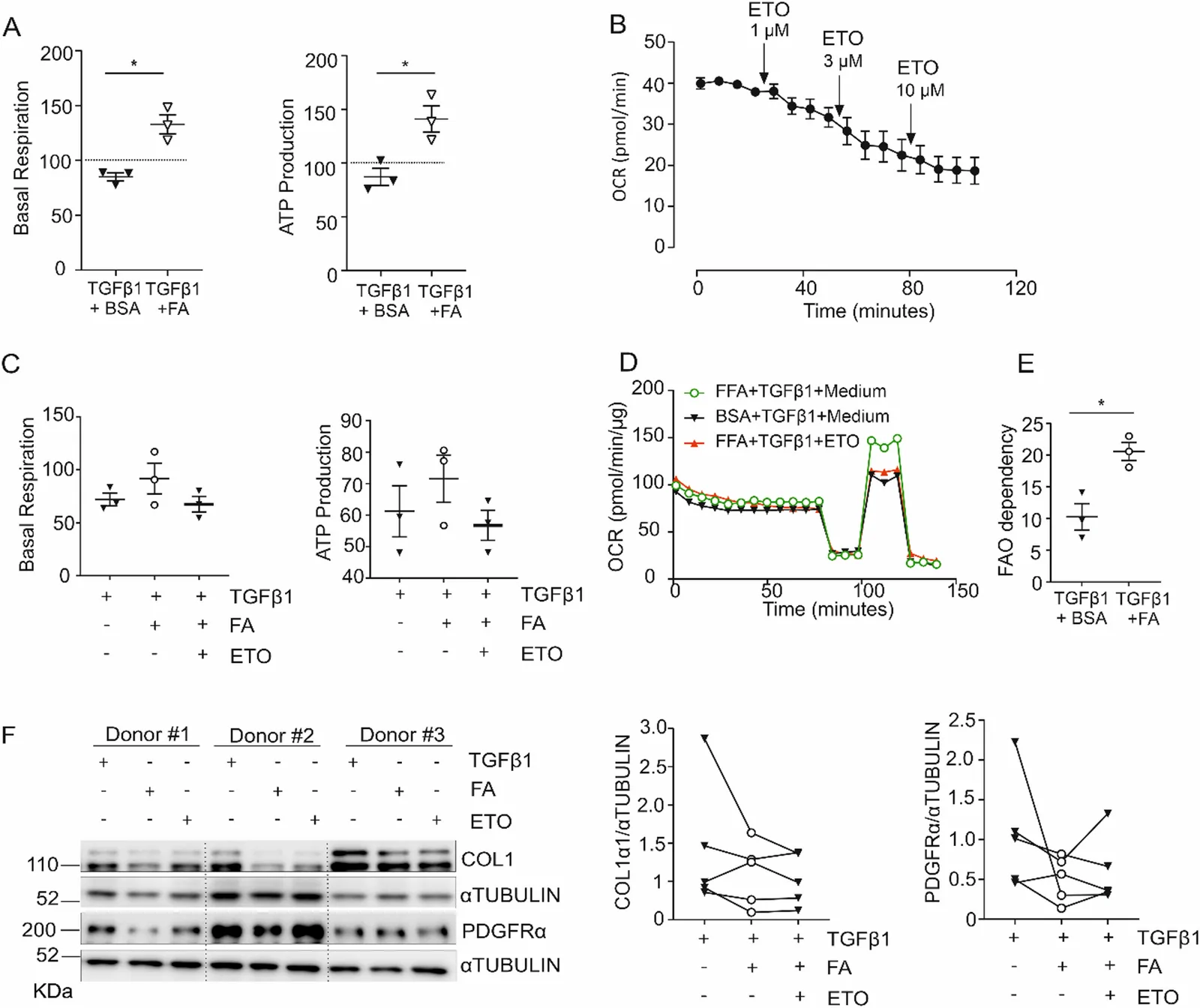
Inhibition of fatty acid oxidation decreases mitochondrial activity but does not restore collagen-I production. (**A**) Basal respiration and ATP production measured by Seahorse analysis in human pulmonary fibroblasts (hPFs) isolated from donor lungs and treated with a fatty acid (FA) mixture (16:0, 18:0, 18:1) for 72 h with or without TGFβ1 (10 ng/ml) (*n* = 3). The dotted line indicates vehicle control (BSA-treated) cells. (**B**) Etomoxir (ETO) dose–response analysis measured by oxygen consumption rate (OCR; pmol/min) using Seahorse analysis in donor hPFs. Concentrations of 1 µM, 3 µM, and 10 µM were tested sequentially. (**C**) Basal respiration and ATP production and (**D**) OCR (pmol/min/µg) measured by Seahorse in hPFs isolated from donor lungs and treated with TGFβ1 (10 ng/ml) with or without FA mixture (16:0, 18:0, 18:1) for 72 h, followed by acute treatment with ETO (4 µM)/Medium prior to measurement (*n* = 4 mixed hPF, 3 technical replicates). (**E**) Fatty acid oxidation (FAO) dependency is derived from the analysed Seahorse measurement depicted in panel D. (**F**) Representative western blot and relative quantification of COL1 and PDGFRα in hPFs isolated from donor lungs and treated with the FA mixture (16:0, 18:0, 18:1) for 48 h with or without TGFβ1 (10 ng/ml), followed by treatment with ETO (4 µM) for 24 h with or without TGFβ1 (10 ng/ml) (*n* = 5). Protein levels were normalized to the loading control α-tubulin. Data are presented as mean ± SEM. Statistical significance for panels A and E was determined using an paired two-tailed Student’s *t*-test, while panel C and F using one-way ANOVA followed by Dunnett’s multiple comparisons test. *P* < 0.05 was considered statistically significant

In order to prove that hPF usage of FA is increased in the concomitant stimulation with TGFβ1 and FA, we performed a seahorse analysis. We observed that FA mixture was able to restore the TGFβ1 induced mitochondrial dysfunction, as shown by enhanced mitochondrial basal respiration and enhanced ATP production (Fig. 6A, dotted line indicates vehicle control cells). To understand if the antifibrotic effect of the FA treatment was dependent of the fatty acid oxidation (FAO), we decided to use a FAO inhibitor, etomoxir (ETO) [39]. After confirming no alteration on cell growth (Supplementary Fig. 7D), decrease OCR (Fig. 6B-D) and AMPK activation as a consequence of changed ADP/ATP ration upon etomoxir (Supplementary Fig. 7E), we analyzed the effect of 4 µM etomoxir on the mitochondrial function of hPF stimulated with TGFβ1 and FA mix. We observed that etomoxir treatment tendentially rescued the enhanced basal respiration and ATP production (Fig. 6C) induced by FA. Importantly, FA mix-TGFβ1 co-treatment statistically enhanced FAO dependency compared to only TGFβ1 stimulation (Fig. 6E). In line with these results, we observed a tendency in lower amount of TGFβ1-induced lactate production in FA-treated donor hPF (Supplementary Fig. 7F). While etomoxir was able to reverse the TGFβ1 induced metabolic shift, it was not able to reverse the TGFβ1 induced COL1 and PDGFRA protein and mRNA level (Fig. 6F and Supplementary Fig. 7G), and LDHA expression level (Supplementary Fig. 7H). Altogether this data suggests that in hPF, available FA are partly stored in form of lipid droplets- similarly to what we observed in *Angptl4* KO mice- and partly used to enhance fatty acid oxidation. In the presence of TGFβ1, exogenous fatty acids attenuate both the metabolic shift and collagen production. However, blocking fatty acid oxidation under these conditions reverses only the metabolic effects, without affecting collagen production, suggesting that fatty acid–mediated regulation of collagen might occur through mechanisms independent of fatty acid oxidation.

### Fatty acid availability suppresses collagen production in IPF-derived fibroblasts

**Fig. 7:**
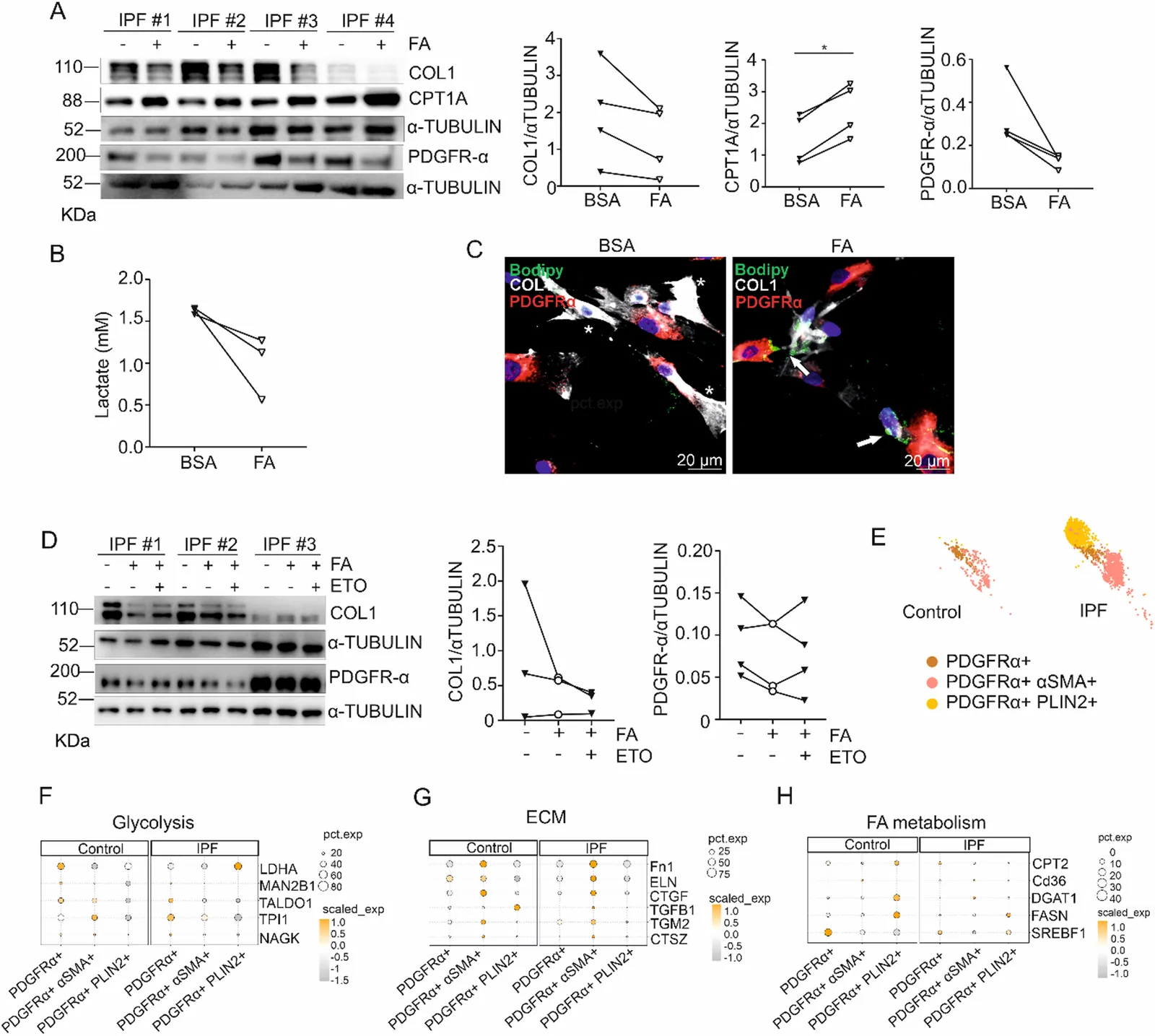
Fatty acid availability decreases collagen production in IPF-derived human pulmonary fibroblasts. (**A**) Representative western blot and relative quantification of COL1, PDGFRα, and CPT1α in human pulmonary fibroblasts (hPFs) isolated from IPF lungs and treated with a fatty acid (FA) mixture (16:0, 18:0, 18:1) for 72 h (*n* = 4). Protein levels were normalized to the loading control α-tubulin. (**B**) Lactate levels (mM) measured in the culture media of hPFs isolated from IPF lungs and treated with the FA mixture (16:0, 18:0, 18:1) for 72 h (*n* = 3). (**C**) Representative images of triple immunofluorescence staining showing COL1 (white), PDGFRα (red), and BODIPY (green) in IPF-derived hPFs treated with the FA mixture (16:0, 18:0, 18:1) for 72 h (*n* = 3–4). Scale bar: 20 μm. Arrows indicate lipid droplet–positive cells, and asterisks indicate collagen-positive cells. (**D**) Representative western blot and relative quantification of COL1 and PDGFRα in hPFs isolated from IPF lungs and treated with the FA mixture (16:0, 18:0, 18:1) for 48 h followed by treatment with etomoxir (ETO; 4 µM) for 24 h (*n* = 4). Protein levels were normalized to the loading control α-tubulin. (**E**) Uniform manifold approximation and projection (UMAP) of human scRNA-seq data (GSE135893) from control (*n* = 10) and pulmonary fibrosis lungs (*n* = 20). Colors represent different fibroblast populations. Dot plots showing genes involved in (**F**) glycolysis, (**G**) extracellular matrix (ECM), and (**H**) fatty acid (FA) metabolism in fibroblast populations from control and IPF lungs. Data are presented as mean ± SEM. Statistical significance for panels A and B was determined using a paired two-tailed Student’s *t*-test. Panel D was determined using using one-way ANOVA followed by Sidak’s multiple comparisons test. *P* < 0.05 was considered statistically significant

To assess the clinical relevance of our finding, we tested the FA mixture on IPF-derived hPF. FA stimulation of hPF did not affect their growth curve (Supplementary Fig. 8A). Contrary to what observed in donor hPF, lower concentration of FA (50 µM and 85 µM) was not able to influence protein level of collagen- I and PDGFRA (Supplementary Fig. 8B) However, 125µM of each FA was able to decrease endogenous *COL1* and *PDGFRA* in and to increase CPT1 protein and mRNA level (Fig. 7A and Supplementary Fig. 8C-D). The FA mixture decreased lactate production of IPF-derived hPF (Fig. 7B) and the expression of the glycolytic gene *GAPDH* (Supplementary Fig. 8E). IPF-hPF displayed lipid droplet accumulation (Fig. 7C- green staining highlighted by the arrow) and increased expression of *ADRP* and *CD36* gene (Supplementary Fig. 8F), suggesting cell storage of the available FA. Blockage of CPT1a with etomoxir was not able to restore the TGFβ1 induced collagen-I production and lactate production, as suggested by the expression of LDHA (Fig. 7H and Supplementary Fig. 8G). To determine whether our findings are reflected in human idiopathic pulmonary fibrosis (IPF) lungs in vivo, we analyzed the expression of genes involved in glycolysis, extracellular matrix (ECM) remodeling, and fatty acid metabolism using a publicly available single-cell RNA-sequencing dataset (GSE135893). Following comprehensive quality control and filtering, *PDGFRα+* fibroblast populations were selected and further stratified into three subsets: single *PDGFRα+* fibroblasts, double *αSMA+/PDGFRα* myofibroblasts, and double *PLIN2+/PDGFRα+* lipofibroblasts (Fig. 7E and Supplementary Fig. 9–10).

As shown in the dot plots, both *αSMA+/PDGFRα+* and single *PDGFRα+* fibroblasts exhibited high expression of glycolysis-associated genes in donor and IPF lungs (Fig. 7F). Among these populations, *αSMA+/PDGFRα+* represented the predominant subtype contributing to extracellular matrix (ECM) gene expression in both donor and IPF samples (Fig. 7G). In contrast, *PLIN2+/PDGFRα+* lipofibroblasts displayed low expression of glycolysis-related genes, with the exception of *LDHA* in IPF lungs (Fig. 7F), and showed minimal expression of ECM-associated genes (Fig. 7G).

Notably, genes associated with fatty acid metabolism were highly enriched in *PLIN2+/PDGFRα+* cells in donor lungs but were markedly reduced in IPF lungs (Fig. 7H), indicating a loss of the fatty acid metabolic signature within this lipofibroblasts population in fibrotic tissue. This shift suggests an altered metabolic program in *PLIN2+/PDGFRα+* fibroblasts during fibrosis.

## Discussion

The present study demonstrates that increased fatty acid (FA) availability exerts a potent anti-fibrotic effect by suppressing collagen production in vitro. We observed that FA provision to fibroblasts induces a rapid accumulation of ADRP, signaling the formation of lipid droplets. Upon co-stimulation with TGFβ1, ADRP expression decreased, suggesting that these lipid stores are actively utilized during the fibrotic response. This metabolic shift was further supported by an increase in CPT1 protein levels, indicating the activation of fatty acid oxidation (FAO). Notably, while FA provision successfully rewired TGFβ1 - induced metabolic alterations via FAO, this increase in oxidation was not responsible for the reduction in collagen production observed upon FA treatment. We confirmed that metabolic interventions and the *Angptl4* genetic depletion reduce bleomycin-induced fibrosis. This effect was most consistently observed when analyzing the entire left lung, whereas analyses of individual lobes yielded greater variability. While it is important to note that the strongest support for reduced fibrosis in this study comes from histomorphometry analysis, our study, together with previous reports [30–32, 35, 57, 58], suggest that larger lung regions provide more representative measurements, with histology offering a comprehensive assessment and biochemical analyses of individual lobes providing less consistent information. Building on the existing evidence of the protective effects of *Angptl4* knockout and rosiglitazone and metformin treatment, we identify enhanced fatty acid availability as the unifying component underlying fibrosis reduction in all three models.

Our data add to a growing body of evidence showing that lipid metabolism is altered during development and progression of lung fibrosis [59]. Neutral lipids are used for many purposes such as production and maintenance of cell membranes, energy provision and storage, and the transmission of cell signaling [60–62]. Triglycerides (TGs), cholesterol, and sterol esters belong to the neutral lipids [60, 63]. TGs are stored in the form of lipid droplets, which are small cytoplasmic organelles consisting of a phospholipid monolayer with the hydrophobic domain inside [60, 63]. In situations of increased energy demand, cells break down the TG in lipid droplets into free fatty acids, which then undergo beta-oxidation leading to ATP production [64]. In bleomycin-induced lung fibrosis, massive local DNA damage occurs first, which initiates the inflammatory response and then the fibrotic process [65, 66]. There is much evidence to suggest that DNA damage can influence metabolism in cancer cells [67, 68]. From a biological point of view, cellular damage has a strong impact on energy production processes, which are activated to fuel the repair mechanisms. In this context, we observed a statistical decrease in circulating fatty acids in wild type mice exposed to bleomycin compared to saline. The lack of fatty acid supply in the lung could be a crucial initiating factor to lung fibrosis. Indeed, we observed less fibrosis in *Angptl4* KO mice subjected to bleomycin compared to their WT counterpart. While WT mice respond to bleomycin by decreasing the amount of circulating fatty acids, *Angptl4* KO mice respond to bleomycin by tendentially enhancing circulating fatty acids. These results suggest that in response to a lung damage *Angptl4* KO mice are able to increase available fatty acids which may promote the resolution process and hence decrease fibrosis. Interestingly we observed that our in vivo models, although targeting different signaling pathways, they all display an upregulation of carnitine palmitoyltransferase 1a (CPT1a) as a common denominator. CPT1a is the rate limiting enzyme of fatty acid oxidation facilitating the transport of fatty acids from the cytosol to the mitochondria to initiate fatty acid oxidation [69]. The activation of CPT1a is mediated, among other mechanism, by substrate availability such as fatty acids, transcriptional activation by PPARγ and inhibition relief by reducing inhibitor molecules such as malonyl-CoA [70]. ANGPTL4 is an inhibitor of the lipoprotein lipase, the enzyme responsible of digesting triglycerides to fatty acids and glycerol [71], indicating ANGPTL4 is an indirect regulator of fatty acids turnover [72]. Rosiglitazone acts as a direct activator of PPARγ signaling [73], while metformin has been reported to activate the energy metabolism regulator 5’-AMP-activated protein kinase (AMPK) [35, 74], an indirect inhibitor of malonyl-CoA [75]. It is clear that the convergence on CPT1a represents a previously unrecognized common node in FAO across these three metabolic interventions in lung fibrosis. Further evidence suggests that CPT1a is important not only for fibroblasts biology but also for type 2 cells (AT2) [76], placing CPT1a and enhanced β oxidation as a central player in the resolution of lung fibrosis. These findings, however, present a conceptual paradox, as our data also suggest that lipid droplet accumulation may exert a protective effect against fibrosis. On one hand we have observed that fibroblasts can uptake and accumulate fatty acids in form of lipid droplets, likely as a protective mechanism against lipotoxicity [77]. On the other hand, the decrease of lipid droplets marker (ADRP) and the increased expression of lipid droplets biosynthesis (DGAT1) with the pro-fibrotic agent TGFβ1, indicates smaller and more unstable lipid droplets that are readily mobilized while still warranting lipotoxicity protection [78, 79]. These findings suggest that enhanced β oxidation is required to accommodate the metabolic shift induced by TGFβ1, but the intracellular lipid reserve remain essential to sustain β oxidation. This process could present a coordinated lipofibroblasts-like differentiation program which includes both storage and oxidation. Interestingly, etomoxir treatment was able to restore the TGFβ1 induced metabolic shift, but not the collagen production. This can be merely a methodological consideration (timing, dosage) [80], could point to alternative metabolic signaling [80] or intracellular signaling of fatty acids which influence collagen production [81, 82] or could suggest that the sole storage of fatty acids is sufficient to decrease matrix production. Suppression of collagen, regardless of β oxidation confirms that FA provision induced antifibrotic state and reveals the multifactorial collagen regulation.

The mechanism by which FA influence collagen production might also be different between fibroblasts sub population as FA supplementation affects differently *PDGFRα+* and *αSMA+* cells. We have previously shown that *PDGFRα* positive cells, together with *αSMA* positive cells, contribute substantially to collagen production [14]. Here in all three in vivo lung models, treatment resulted in a preferential—though not exclusive—reduction in PDGFRα protein levels compared to αSMA. In the in vitro settings, *αSMA* positive cells decreased collagen production while *PDGFRα* positive cells decreased in number upon FA stimulation. These results suggest a dual role of FA in different fibroblasts. While the effect on αSMA positive cells seem to be a direct effect on collagen production, the additional effect on *PDGFRα* positive cells is novel and point towards a cell identity effect. This could explain the inconsistency in change of PDGFRα protein expression in vivo and in vitro. It is plausible that in vivo FA act on other cell types, such as type 2 epithelial cells [83]. However, it is well established that PDGFRα-positive cells can give rise to both pro-fibrotic fibroblasts and lipofibroblasts [84] which might respond differently to FA. It is possible that the pro-fibrotic phenotype of PDGFRα-positive cells is driven by a switch from β-oxidation to glycolysis. Indeed, Cong Ran et al. associated PDGFR and/or PDGF ligand with increase glycolytic capacity [85] while another study reported TGFβ1-induced activation of glycolysis mediated by PDGF signaling [86]. Our results corroborate these findings, as fatty acid stimulation was able to decrease lactate production and genes involved in glycolysis. Together, these data support the recent hypothesis of a metabolic shift in lung fibrosis fibroblasts - from fatty acid metabolism to glycolysis [87], further confirmed by recent studies reporting TGFβ1 as an activator of glycolysis [17, 88]. In line with this notion and with our results, a recent publication showed that in silica induced fibrosis, another type of lung fibrosis, benefit from FA oxidation activation and glycolysis inhibition resulting in less activated fibroblasts and less collagen [89]. Intriguingly, we showed in this study that *PLIN2/PDGFRα+* cells (lipofibroblasts) do express a marker of anaerobic glycolysis in IPF while they are rich in FA metabolism gene in donor lungs. This gene signature can be integrated with the enhanced *CPT1a*, *CD36*, *ADRP* gene expression observed in our FA stimulated cells. Although these changes may partly reflect the simplified nature of the in vitro system, our results suggest that FA stimulation on cell cultured fibroblasts may represent the in vitro counterpart of the lipofibroblasts population. Additionally, these results also suggest that lipofibroblasts may be the fibroblast subpopulation that benefits most from the enhancement of fatty acid metabolism and thus initiates the resolution process. Whether this process can be reactivated at advanced stage of lung fibrosis, when the disease is often diagnosed, remains an unanswered question. Although the mechanism requires further research and these results cannot be directly translated into clinical practice, this study suggests that prolonged impairment of fatty acid may promote the development of lung fibrosis. These findings not only strengthen the link between metabolic changes and fibrosis development, but also position fatty acid availability as a key mechanistic target in pulmonary fibrosis.

## Supplementary Information

Below is the link to the electronic supplementary material.

Supplementary File 1 Bleomycin treatment decreases expression of fatty acid metabolism genes. Gene expression analysis of (**A**) Acsl1 and (**B**) Srebf1 performed in lung homogenates from WT mice treated with bleomycin or saline and sacrificed 14 days post-treatment (n = 10–15). Data are presented as mean ± SEM. Statistical significance was determined using an unpaired two-tailed Student’s t-test. P < 0.05 was considered statistically significant. (PNG 60.9 KB)

High Resolution Image (TIF 10.9 MB)

Supplementary File 2 Common downregulated genes between the Pdgfrα and αSma positive cells after bleomycin administration. Venn diagram showing significantly downregulated genes shared between Pdgfrα+ and αSma+ cells. (PNG 124 KB)

High Resolution Image (TIF 13.7 MB)

Supplementary File 3 Metabolic interventions decrease collagen-I expression but do not improve lung compliance. (A–B) Gene expression analysis of Col1a1 in (**A**) rosiglitazone (Ro)-treated mice (n = 8) and (**B**) metformin (Met)-treated mice (n = 9–11) 14 days after bleomycin administration. (C–E) Lung compliance (Cst) analysis in (**C**) Angptl4 knockout (KO) mice (n = 10–15), (**D**) rosiglitazone-treated mice (n = 8), and (**E**) metformin-treated mice (n = 9–11), measured 14 days after bleomycin administration. Data are presented as mean ± SEM. Statistical significance was determined using an unpaired two-tailed Student’s t-test. P < 0.05 was considered statistically significant. (PNG 160 KB)

High Resolution Image (TIF 38.9 MB)

Supplementary File 4 Metabolic interventions do not alter αSMA or pro-SPC protein levels. Western blot analysis of αSMA and pro-SPC (SFTPC) and relative quantification of αSMA in (**A**) Angptl4 knockout (KO) and WT mice (n = 5), (**B**) rosiglitazone (Ro)-treated mice (n = 7), and (**C**) metformin-treated mice (n = 7) subjected to bleomycin administration and analyzed 14 days post-treatment. Protein levels were normalized to the loading control α-tubulin. Data are presented as mean ± SEM. Statistical significance was determined using an unpaired two-tailed Student’s t-test. P < 0.05 was considered statistically significant. (PNG 626 KB)

High Resolution Image (TIF 35.8 MB)

Supplementary File 5 Fatty acids do not influence donor fibroblast growth. Human pulmonary fibroblasts (hPFs) isolated from donors (n = 6) were treated with a fatty acid (FA) mixture (16:0, 18:0, 18:1) for 48 h. Live-cell microscopy images were acquired hourly from five regions of each well to assess cell number. Cell growth prior to treatment was set to 100%. BSA was used as the vehicle control, and two FA mixture concentrations were tested (125 μM:125 μM:125 μM and 250 μM:250 μM:250 μM). Data are presented as mean ± SEM. Statistical significance was determined using Two Way ANOVA. P < 0.05 was considered statistically significant. (PNG 82.4 KB)

High Resolution Image (TIF 13.7 MB)

Supplementary File 6 Fatty acids do not negatively affect hPF viability or induce ER stress. (**A**) Gene expression analysis of COL1A1 in human pulmonary fibroblasts (hPFs) isolated from donors (n = 4) treated with TGFβ1 (10 ng/ml) alone or in combination with a fatty acid (FA) mixture (16:0, 18:0, 18:1) for 72 h.(**B**) Western blot analysis and relative quantification of COL1 in hPFs isolated from donors (n = 3) treated with BSA, FAs mix (16:0, 18:0, 18:1) alone, TGFβ1 (10ng/mL) alone or in combination with FAs mix (16:0, 18:0, 18:1) for 72 h at two concentrations (50 μM:50 μM:50 μM and 85 μM:85 μM:85 μM). Protein levels were normalized to the loading control α-tubulin. (**C**) Gene expression analysis of CPT1α in hPFs isolated from donors (n = 3) treated with TGFβ1 (10ng/mL) alone or in combination with FAs mix (16:0, 18:0, 18:1) for 72 h. (**D**) Western blot analysis and relative quantification of BiP in hPFs isolated from donors (n = 6) treated with BSA, FAs mix (16:0, 18:0, 18:1) alone, TGFβ1 (10ng/mL) alone or in combination with FAs mix (16:0, 18:0, 18:1) for 72 h. Protein levels were normalized to the loading control α-tubulin. (**E**) LDH cytotoxicity assay performed in hPFs isolated from donors (n = 3) treated with TGFβ1 (10ng/mL) alone or in combination with FAs mix (16:0, 18:0, 18:1) for 72 h. Data are presented as mean ± SEM. Statistical significance was determined using a paired two-tailed Student’s t-test for panel A-D, while panel E was determined using ONE Way ANOVA followed by Sidak’s multiple comparisons test. P < 0.05 was considered statistically significant. (PNG 361 KB)

High Resolution Image (TIF 87.5 MB)

Supplementary File 7 Fatty acid oxidation drives metabolic reprogramming in human pulmonary fibroblasts. (A–C) Gene expression analysis of (**A**) DGAT1, (**B**) HSL, and (**C**) ATGL in human pulmonary fibroblasts (hPFs) isolated from donors (n = 4) treated with a fatty acid (FA) mixture (16:0, 18:0, 18:1) with or without TGFβ1 (10 ng/ml) and collected at different time points (15 h, 24 h, and 48 h). (**D**) hPFs isolated from donors (n = 4) treated with etomoxir (ETO; 4 µM) for 24 h. Live-cell microscopy images were acquired hourly from five regions of each well to assess cell number. Cell growth prior to treatment was set to 100%. Normal culture medium (NT) was used as the control condition. (**E**) Western blot analysis of phospho-AMPK (pAMPK) and total AMPK in hPFs isolated from donors (n = 3) treated with TGFβ1 (10 ng/ml) and ETO for 3 h, 8 h, and 24 h. α-tubulin was used as the loading control. (**F**) Lactate levels (mM) measured in the culture media of hPFs isolated from donors (n = 4) treated with the FA mixture (16:0, 18:0, 18:1) with or without TGFβ1 (10 ng/ml).(G–H) Gene expression analysis of (**G**) COL1A1 and (**H**) LDHA in hPFs isolated from donors (n = 5) treated with the FA mixture (16:0, 18:0, 18:1) for 48 h with or without TGFβ1 (10 ng/ml), followed by treatment with ETO (4 µM) in the presence of TGFβ1 for 24 h. Data are presented as mean ± SEM. Statistical significance for panels A–C was determined using an unpaired two-tailed Student’s t-test. Panel D using Two Way ANOVA while panels F–H were analyzed using one-way ANOVA followed by Sidak’s multiple comparisons test. P < 0.05 was considered statistically significant. (PNG 620 KB)

High Resolution Image (TIF 96.1 MB)

Supplementary File 8 Fatty acid treatment induces metabolic changes in end-stage IPF-derived human pulmonary fibroblasts. (**A**) Growth of human pulmonary fibroblasts (hPFs) isolated from end-stage IPF lungs (n = 4) treated with a fatty acid (FA) mixture (16:0, 18:0, 18:1) for 48 h. Live-cell microscopy images were acquired hourly from five regions of each well to assess cell number. Cell growth prior to treatment was set to 100%. BSA was used as the vehicle control. (**B**) Western blot analysis and relative quantification of COL1 in hPFs isolated from IPF lungs (n = 3) treated with the FA mixture (16:0, 18:0, 18:1) for 72 h at two concentrations (50 μM:50 μM:50 μM and 85 μM:85 μM:85 μM). Protein levels were normalized to the loading control α-tubulin.(C–D) Gene expression analysis of (**C**) COL1A1 (n = 7) and PDGFRα (n = 6), and (**D**) CPT1α (n = 6), GAPDH (n = 4), CD36 (n = 6), and ADRP (n = 6) in IPF-derived hPFs treated with the FA mixture (16:0, 18:0, 18:1) for 72 h. (**E**) Gene expression analysis of LDHA in IPF-derived hPFs (n = 4) treated with the FA mixture (16:0, 18:0, 18:1) for 48 h followed by etomoxir (ETO; 4 µM) treatment for 24 h. Data are presented as mean ± SEM. Statistical significance for panel A was determined using Two Way ANOVA. Panel B–D was determined using a paired two-tailed Student’s t-test, while panel E was analyzed using a repeated-measures one-way ANOVA followed by Sidak’s multiple comparisons test. P < 0.05 was considered statistically significant. (PNG 229 KB)

High Resolution Image (TIF 78.3 MB)

Supplementary File 9 Quality control and batch correction of the human lung scRNA-seq dataset (GSE135893). (**A**–**C**) Pre–quality control (Pre-QC) plots showing (**A**) library size (total counts), (**B**) number of unique genes detected, and (**C**) percentage of mitochondrial gene counts. (**D**–**F**) Post–quality control (Post-QC) plots showing (**D**) library size, (**E**) number of unique genes detected, and (**F**) percentage of mitochondrial gene counts after filtering. (**G**–**J**) Uniform manifold approximation and projection (UMAP) of the human scRNA-seq dataset (GSE135893) following quality control and batch correction. Cells are coloured by (**G**) processing site and (**H**) flowcell, demonstrating an even cell distribution without pronounced batch effects. Cells are further annotated by (**I**) sample ID and (**J**) library ID. (PNG 1.22 MB)

High Resolution Image (TIF 143 MB)

Supplementary File 10 Selected genes in the human lung scRNA-seq dataset (GSE135893). (**A**–**D**) Uniform manifold approximation and projection (UMAP) of the human scRNA-seq dataset (GSE135893). (**A**) UMAP displaying expression of canonical lung cell markers. (B–D) PDGFRα⁺ fibroblasts from control and IPF samples were extracted and re-clustered to identify distinct fibroblast populations, including cells expressing (**B**) myofibroblast markers, (**C**) PLIN2⁺ fibroblast markers, and (**D**) other fibroblast markers. (**E**) Boxplots showing the number of cells in each fibroblast subset (fibroblasts, myofibroblasts, and PLIN2⁺ fibroblasts). Each dot represents the number of cells of a given fibroblast subset detected in one patient/sample. Boxes indicate the median and interquartile range. (PNG 620 KB)

High Resolution Image (TIF 114 MB)

Supplementary File 11 Marker strategy used to define fibroblast subpopulations. Fibroblast subpopulations were defined based on marker expression as follows: PDGFRα⁺ fibroblasts, PDGFRα⁺αSMA⁺ myofibroblasts, and PDGFRα⁺PLIN2⁺ lipofibroblasts. This classification was applied to both control and IPF samples. (PNG 128 KB)

High Resolution Image (TIF 25.3 MB)

Supplementary Material 12

## Data Availability

The datasets generated analysed during the current study are available in the NCBI repository under corresponding links: GSE135893 (https://www.ncbi.nlm.nih.gov/geo/query/acc.cgi?acc=GSE135893), GSE201698 (https://www.ncbi.nlm.nih.gov/geo/query/acc.cgi?acc=GSE201698), GSE126205 (https://www.ncbi.nlm.nih.gov/geo/query/acc.cgi?acc=GSE126205). Figures were designed in the CorelDRAW^®^ Graphics Suite 2024. The graphical method abstract in Fig. 2 was produced using BioRender.

## Abbreviations

16:0: Palmitic acid
18:0: Stearic acid
18:1: Oleic acid
αSMA: Alfa smooth muscle actin
β2M: Beta-2-microglobulin
ΔCT: Threshold cycle difference
A4galt: 1,4-galactosyltransferase
Aacs: Acetoacetyl-CoA synthetase
Abdh12: Lysophosphatidylserine lipase
Acaa2: Acetyl-CoA Acyltransferase 2
Acsl1: Acyl-CoA synthetase long-chain family member 1
ACTA2 +: Myofibroblasts; alpha-smooth muscle actin-positive fibroblasts
ANGPTL4: Angiopoietin like 4
ANGPTL4 KO: Angiopoietin – like 4 knockout
ApoE: Apolipoprotein E
BALF: Broncho-alveolar lavage fluid
BW: Body weight
Cd36: Fatty acid Translocase
CPT1a: Carnitine palmitoyltransferase 1 A
COL1A1: Collagen 1 alfa 1
Dgat2: Diacylglycerol O-acyltransferase 2
ECAR: Extracellular acidification rate
ECM: Extracellular matrix
ETO: Etomoxir
FA: Fatty acids
Fasn: Fatty acid Synthase
hPF: Human parenchymal fibroblasts
IPF: Idiopathic pulmonary fibrosis
LdhA: Lactate Dehydrogenase A
LPL: Lipoprotein lipase
OCR: Oxygen consumption rate
PBGD: Porphobilinogen deaminase
PDGFRα+: Platelet-derived growth factor receptor alpha-positive
pro-SPC: Pro Surfactant Protein C
PVDF: Polyvinylidene fluoride membranes
Srebpc: Sterol Regulatory Element-Binding Protein 1c
TGFβ1: Transforming Growth Factor Beta 1
WT: Wild-type
