# Supplementary material for: Enhanced fatty acid availability rewires fibroblasts metabolism and decreases collagen deposition in lung fibrosis
Source: Cell Mol Life Sci. 2026 Jun 1;83(1):317. doi: 10.1007/s00018-026-06256-4 (PMC13518688; doi:10.1007/s00018-026-06256-4)
Supplement: Supplementary file 1 [file 18_2026_6256_MOESM12_ESM.docx]

**Supplementary Table 1: Primer sequence.**

| **Gene** | **Sequence** |
| --- | --- |
| hADRP_fwd | AGTCTTGGGGAGTCGGAT |
| hADRP_rev | CACGGGAGTGAAGCTTGGTA |
| hDGAT1__fwd | CAACTACCGTGGCATCCTG |
| hDGAT1_rev | TTCTCCAGAAATAACCGGGC |
| hATGL_fwd | AACCTCTACCGCCTCTCC |
| hATGL_rev | GGTATCCCTGCTTGCACATC |
| hHSL_fwd | TGTCACGCTACACAAAGGCT |
| hHSL__rev | GGTCACACTGAGGCCTGTC |
| hB2M_fwd | CCTGGAGGCTATCCAGCGTACTCC |
| hB2M_rev | TGTCGGATGGATGAAACCCAGACA |
| hPBGD_fwd | CTGCAACGGCGGAAGAAAA |
| hPBGD_rev | AATCTTGTCCCCTGTGGTGG |
| hCOL1A1_fwd | ACATGTTCAGCTTTGTGGACC |
| hCOL1A1_rev | TGTACGCAGGTGATTGGTGG |
| hCOL3_fwd | GGTGTCCCAGGGAAAGATGG |
| hCOL3_rev | CTCTCTCACCAGGGCTACCA |
| hGAPDH_fwd | CCACATCGCTCAGACACCAT |
| hGAPDH_rev | AAAAGCAGCCCTGGTGACC |
| hCD36_fwd | CAGGTCAACCTATTGGTCAAGCC |
| hCD36_rev | GCCTTCTCATCACCAATGGTCC |
| hCPT1A_fwd | GATCCTGGACAATACCTCGGAG |
| hCPT1A_rev | CTCCACAGCATCAAGAGACTGC |
| hLDHA_fwd | GGATCTCCAACATGGCAGCCT |
| hLDHA_rev | AGACGGCTTTCTCCCTCTTGC |
| mB2m_fwd | CGGCCTGTATGCTATCCAGAAAACC |
| mB2m_rev | TGTGAGGCGGGTGGAACTGTG |
| mPbgd_fwd | TCCGGAGGCGGGTGTTGAGG |
| mPbgd_rev | GCCAGAGAAAAGTGCCGTGGG |
| mCol1A1_fwd | AACGGGTCCCCTTGGGCCTT |
| mCol1A1_rev | AATGGCACGGCTGTGTGCGA |
| mGapdh_fwd | GGAGAGTGTTTCCTCGTCCC |
| mGapdh_rev | ATGAAGGGGTCGTTGATGGC |
| mCd36_fwd | GCTGTTCTAGAAGTTGCTGCC |
| mCd36_rev | TGAGACTCTGAAAGGATCAGCAC |
| mCpt1a_fwd | GGCATAAACGCAGAGCATTCCTG |
| mCpt1a_rev | CAGTGTCCATCCTCTGAGTAGC |
| mLdha_fwd | CCTGCACCAAACATGCCTA |
| mLdha_rev | CACTGGAGCCAGGTTATACGA |
| mDgat2_fwd | GAAAGGTGGCAGGAGATCG |
| mDgat2_rev | CCAGCTGGATGGGAAAGTAG |
| mFasn_fwd | GGCCCCTCTGTTAATTGGCT |
| mFasn_rev | GGATCTCAGGGTTGGGGTTG |
| mAcsln1_fwd | TGGGGTGGAAATCATCAGCC |
| m Acsln1_rev | CACAGCATTACACACTGTACAACG |
| mSrebf_fwd | GGAGCCATGGATTGCACATT |
| mSrebf_rev | GGCCCGGGAAGTCACTGT |
